# Alpha and beta diversity patterns of macro-moths reveal a breakpoint along a latitudinal gradient in Mongolia

**DOI:** 10.1038/s41598-021-94471-3

**Published:** 2021-07-22

**Authors:** Khishigdelger Enkhtur, Gunnar Brehm, Bazartseren Boldgiv, Martin Pfeiffer

**Affiliations:** 1 Department of Biogeography, University of Bayreuth, Universitätsstraße 30, 95447 Bayreuth, Germany; 2 Phyletisches Museum, Institut für Zoologie und Evolutionsbiologie, Friedrich‐Schiller‐Universität, Vor dem Neutor 1, 07743 Jena, Germany; 3 Ecology Group, Department of Biology, National University of Mongolia, Ikh Surguuliin Gudamj 1, Ulaanbaatar, 14201 Mongolia; 4 Academy of Natural Sciences of Drexel University, Philadelphia, PA 19103 USA

**Keywords:** Biodiversity, Biogeography, Community ecology, Grassland ecology, Macroecology, Entomology

## Abstract

Little is known about the diversity and distribution patterns of moths along latitudinal gradients. We studied macro-moths in Mongolia along an 860 km latitudinal climatic gradient to gain knowledge on community composition, alpha, beta, and gamma diversity as well as underlying factors, which can be used as baseline information for further studies related to climate change. We identified 236 species of moths of ten families. Our study shows that the diversity of moths increased with the latitude, i.e., low species richness in the south and higher richness in the north. Moth community composition changed along the gradient, and we revealed a breakpoint of beta diversity that divided grassland and desert communities. In the desert, beta diversity was driven by species loss (i.e., nestedness), and few tolerant species existed with high abundance. In contrast, in the grassland, beta diversity was driven by species replacement with more unique species, (i.e., species which occurred only in one site). We found the lowest species diversity in the transitional zones dominated by few generalist species such as *Agrotis ripae* and *Anarta trifolii.* Low precipitation and an increasing number of grazing goats are drivers of species loss. We suggest different conservation strategies regarding the contrasting patterns of beta diversity in desert and grassland.

## Introduction

Biodiversity loss has become a pressing global issue in the last decades^1^. Since biodiversity is crucial to maintain ecosystem functions, it is important to study the distribution of organisms and their response to climate change and human disturbance. Recently, a preponderance of studies reported strong declines in insect diversity^2–5^. For example, in Germany’s protected areas flying insect biomass declined by more than 75% within only 27 years, however, the cause is still unclear^6^.

As Simmons et al.^3^ stated, some “global” studies on insect decline should be cautiously interpreted because results based on particular locations do not represent a global scale. Robust insect diversity data representing all major biomes of the world are required^7^. However, data availability is strongly biased across the world towards Europe and North America, especially regarding systematically collected long-term data. Tropical regions are poorly studied. The same is true for the most parts of central and eastern Asia, especially in regard to the diversity and distribution patterns of moths in eastern Russia, northern China and Mongolia. During a previous literature review of studies on geometrid moths, we found that long-term data were unavailable from these regions^8^. This study is an important ”puzzle piece” in filling this gap for future research.

There are approximately 1550 species of Lepidoptera reported in Mongolia^9^; however, there is no complete checklist available. In geometrid moths, a recent checklist reported 388 observed species, but species richness was estimated to be 663 ± 56^8^. Recently, 21 new species have been recorded from western and central Mongolia^10^ and the family Alucitidae was first time reported for Mongolian fauna in 2015 in the Mongolian Altai Mountains^11^. Moreover, several species new for the fauna of Mongolia were reported in Sphingidae, Noctuidae, Cossidae, and Ypsolophidae^12–16^. In the Global Biodiversity Information Facility (GBIF), 919 species of 30 families of Lepidoptera are recorded for Mongolia^17^. This is certainly an underestimate, and not all occurrence data in the literature have been uploaded in GBIF. To summarize, data have been collected incompletely, non-continuously with different efforts, at specific locations, published, and scattered in the literature, thus rendering it impossible to investigate the changes of moth diversity at temporal and spatial scales.

In response to this need, our study focuses on moth diversity and species composition across a latitudinal gradient. Biodiversity across latitudinal gradients is especially important to study as they are the largest and strongest climatic gradients globally. Alpha diversity is the diversity of local communities, while beta diversity is the spatial change in composition between local communities^18^. Beta diversity links alpha and gamma diversity, i.e., large-scale diversity. To measure alpha diversity, we used Hill numbers: species richness, Shannon diversity and Simpson diversity. Hill numbers are a linear measure of diversity, which traditional indices are not, they have the same units and are comfortable to compare sites^19,20^. They account for different levels of diversity and mirror species richness and evenness.

Measuring alpha diversity is vital for conservation purposes since it quantifies the biodiversity of a particular habitat through the baseline measure of species presence and abundance within a local community. Species richness (number of species present) of moths can reflect habitat quality and be an indicator of species sensitivity to environmental changes^21,22^. Pronounced declines of species richness along the latitudinal gradient from the equator to the poles have been demonstrated for almost all taxa in different regions of the world^23–26^. This general trend of declining diversity and richness across latitudes is accompanied by environmental factors such as temperature along altitudinal gradients, land use, and precipitation^27^. As precipitation increases with latitude in most parts of Central Asia^27,28^, this could regionally superimpose patterns of moth richness and diversity patterns.

One crucial question is how species composition changes along latitudinal gradients, i.e., whether the change is due to species replacement or species loss/gain. Different types of measures for beta diversity are available^29–32^. We applied the widely used method by Baselga et al.^33^, which partitions beta diversity into turnover and nestedness. Doing so enables us identifying the leading causes for the differentiation and is further useful for implementing better conservation strategies. Turnover reflects the process of environmental filtering, while nestedness reflects colonization, such as the effects of a lack of available resources^34^.

In Mongolia, on the one hand, a latitudinal or climatic gradient can be one type of environmental filtering. Mongolia is located between 41°35′ and 52°06′ N. This climatic gradient is characterized by higher rainfall and lower temperature in the north and lower precipitation and higher temperature in the south^35^.

On the other hand, grazing patterns represent another type of environmental filtering. In Mongolia, the dominant land use type in the country is free-ranging livestock grazing, thus overgrazing can be the cause of colonization or extinction from one habitat to another^36^. Recently, the number of livestock is increasing, and nowadays, herders tend to be more sedentary than former herders, which causes local to regional pasture degradation. Moreover, the effects of climate change and overgrazing are accelerating each other in a positive feedback loop^36^.

We tested the hypothesis that species diversity and species richness declines with latitude in Mongolia. Moreover, we hypothesized that precipitation positively influences diversity and richness, and that (over-) grazing negatively influences diversity and richness. These (and possibly other environmental variables) could regionally superimpose the expected large-scale latitudinal patterns, resulting in inverse latitudinal gradient patterns and/or breakpoints.

In addition, we investigated (without an a priori hypothesis), how moth species composition or beta diversity differed between sites, and if beta diversity was mainly influenced by spatial turnover (species replacement) or nestedness (species loss or gain). Moreover, our study provides new data on the regional species pool of Mongolia, i.e., how many and which moth species are present, and explores the gamma diversity of moths in Mongolia.

This is the first comprehensive study on macro-moths over large geographic scales in Mongolia and it forms the baseline for future studies. It is necessary to gain knowledge of moth diversity and distribution patterns at local and large-scale level (i.e., alpha and gamma diversity) and how local diversities are organized and vary at large-scale (beta diversity)^37,38^ to develop an effective conservation strategy for the Mongolian moth species and their habitats. Different conservation strategies are required depending on the beta diversity patterns (nestedness or turnover). For the areas with species loss, it is recommended to protect certain species-rich sites; in contrast, for the areas with species replacement, several large different types of sites are needed to be protected^33^. Species-poor sites usually hold only a subset of species-rich sites^39^. In a study of birds and snails, habitat homogeneity was responsible for the nestedness of the animal communities^40,41^. However, it must be noted that habitat simplification can reduce local species richness, and the whole community would be similar, leading to homogenization^42,43^. Thus, it is vital to see both, the smaller more detailed picture as well as the bigger picture in order to consider the fragmentation between the sites and successfully implement conservation plans, both locally and regionally. If the temperature keeps rising and livestock numbers keep increasing, even species-rich sites would be transformed into species-poor sites, making the whole community unable to sustain itself. Some species will disappear due to the loss of suitable habitat, and only species which have tolerance to the disturbance will be left^39,44^. Moreover, in the face of climate change northern sites have the potential of becoming more similar to current day ecological conditions in southern sites. This could lead to homogenization, resulting in a less diverse assemblage. By tracking moth biodiversity along a latitudinal gradient, this study is using a space-for-time substitution (e.g., southern sites could predict future results for northern sites). Thus, our results not only provide necessary baseline reference data, but also essential insights on the future of biodiversity change in a warming world.

## Results

### Alpha diversity

**Figure 1:**
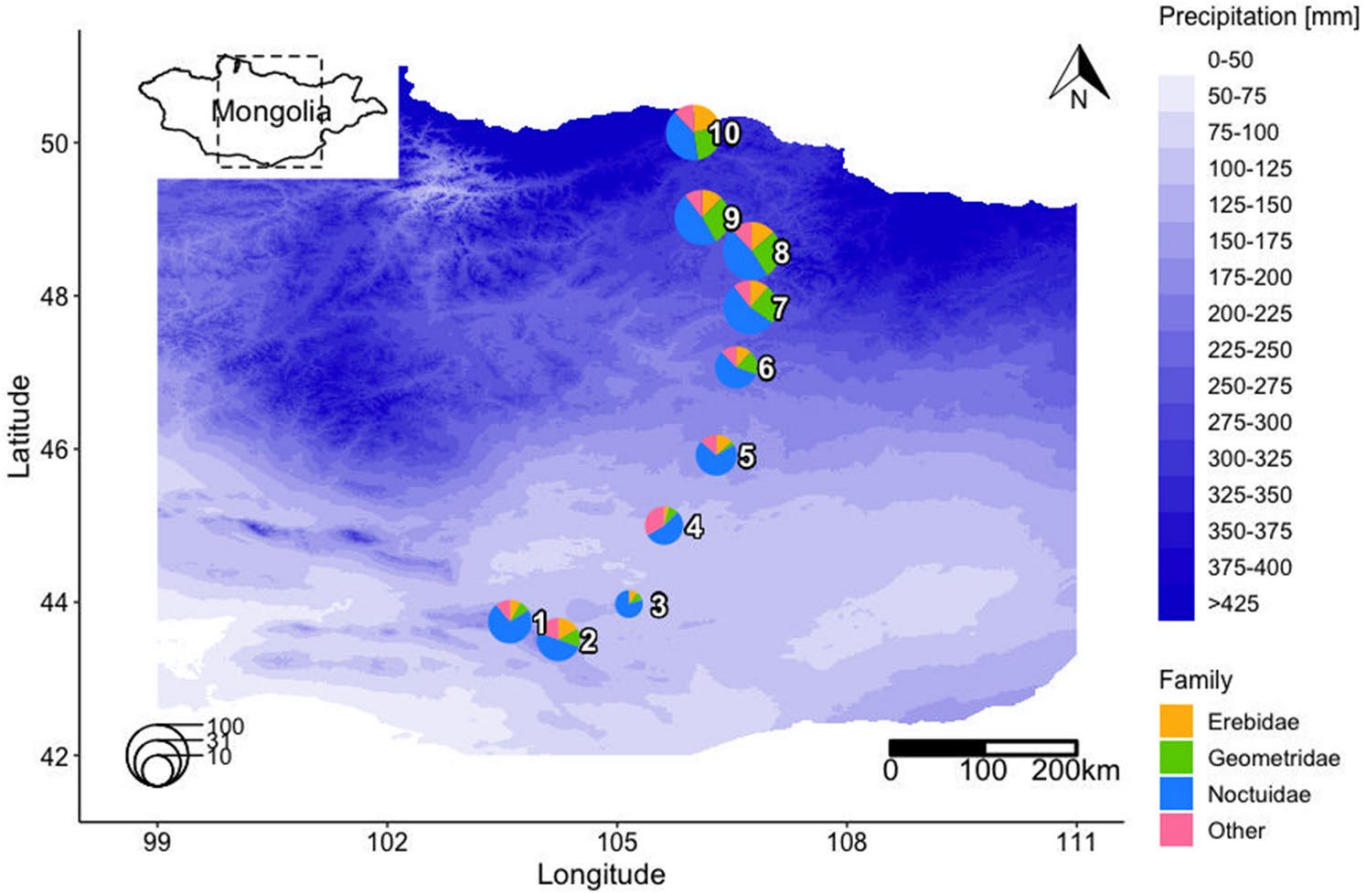
Study area with pie charts showing the percentage species composition of the main moth families: Erebidae, Geometridae, Noctuidae and all other families along the precipitation gradient. In group *Other:* These families are combined: Cossidae, Drepanidae, Lasiocampidae, Notodontidae, Sesiidae, Sphingidae, and Zygaenidae. Pie sizes correspond to species richness of the site (legend on the lower left side) See species richness and diversity of each site in Table S3 in supplementary material. Figure was produced using R software (version 3.6.3, R Core Team, https://www.r-project.org/).

In total, we caught 11,115 macro-moth individuals of 236 species of ten families: 7 Cossidae, 3 Drepanidae, 35 Erebidae, 58 Geometridae, 6 Lasiocampidae, 108 Noctuidae, 7 Notodontidae, 1 Sesiidae, 10 Sphingidae, and 1 Zygaenidae (see the full species list in Table S2 in the supplementary material). Estimated species richness was 461 (iChao1, SE: 22.96, lower 95%: 392, upper 95%: 581), therefore, our samples cover 51% of the estimated species richness. The three most species-rich families were Noctuidae (45.8% of species), Geometridae (24.6%) and Erebidae (14.8%) (Fig. 1). The other families together constituted 14.8% of all species and we combined them into one group (“Other”).

Noctuidae had the highest abundance represented with 8839 specimens, with the commonest species *Agrotis ripae* Hübner, with 5986 individuals collected at nine out of ten sites, especially dominating the sites in the desert. Moth family composition patterns changed along the latitudinal gradient. In the grassland sites, Erebidae, Geometridae, and Noctuidae (and “Other”) shared similar proportions whereas Noctuidae heavily dominated in all desert sites (Fig. 1). K-means clustering separated all sites into two groups of southern “desert” (1–5) and northern “grassland” (6–10) sites (see scree plot in Fig. S1 in supplementary material).

**Figure 2:**
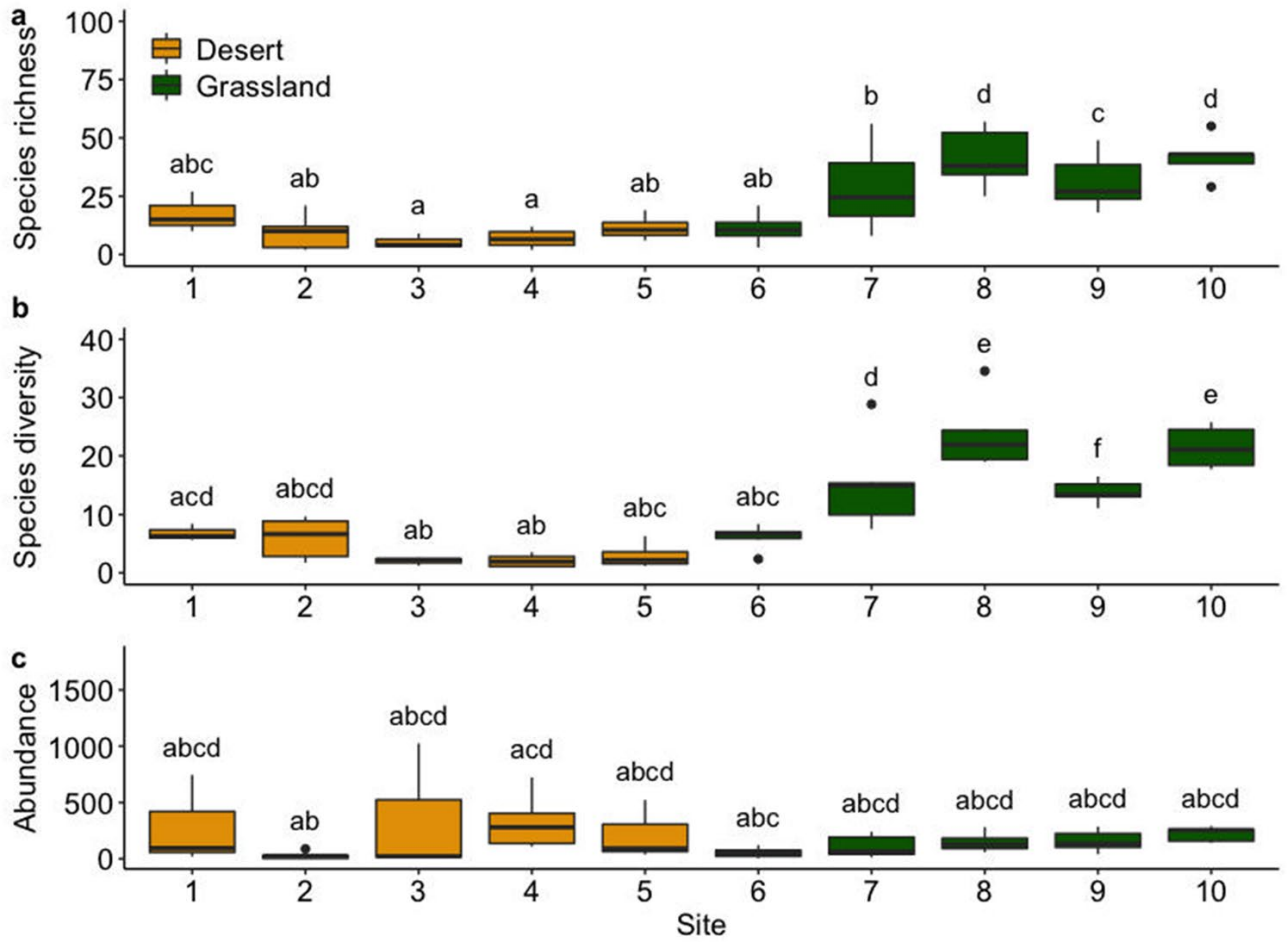
Species richness, Shannon diversity and abundance of ten sites along the latitudinal gradient. Diversity metrics were compared with Wilcoxon test based on the sampling nights of each site. Different letters show significant differences between sites. See the further comparison of species richness, species diversity and abundance at the family level in Fig. S2, Tables S4, S5 and S6 of supplementary material.

Overall, moth species richness (Fig. 2a), species diversity (Fig. 2b), and abundance (Fig. 2c), of the grassland sites (6–10) were significantly higher (*p* < 0.005) than those of the desert sites (1–5). Among the desert sites, species diversity at Site 2 was higher than in all other sites. The most species-rich site was site 8 (grassland), and the most species poor-site was Site 3 (desert). We investigated which functional group of vegetation was responsible for high species richness of moths. As a result of GLM, *forb* impacted the species richness of moths (LM: *R*^2^ = 0.55, *p* = 0.012).

Hill numbers were positively correlated with precipitation and forb cover, and negatively correlated with temperature, wind and number of goats (Table 1).

**Table 1:** Pearson correlation coefficients of Hill numbers with environmental variables.

Hill numbers	Precipitation	Temperature	Forb cover	Goat number	Wind
Species richness	0.92***	− 0.76***	0.99***	− 0.75***	− 0.91***
Shannon diversity	0.89***	− 0.73***	0.96***	− 0.80***	− 0.87***
Simpson diversity	0.92***	− 0.76***	0.92***	− 0.75***	− 0.88***

### Species abundance and richness pattern

**Figure 3:**
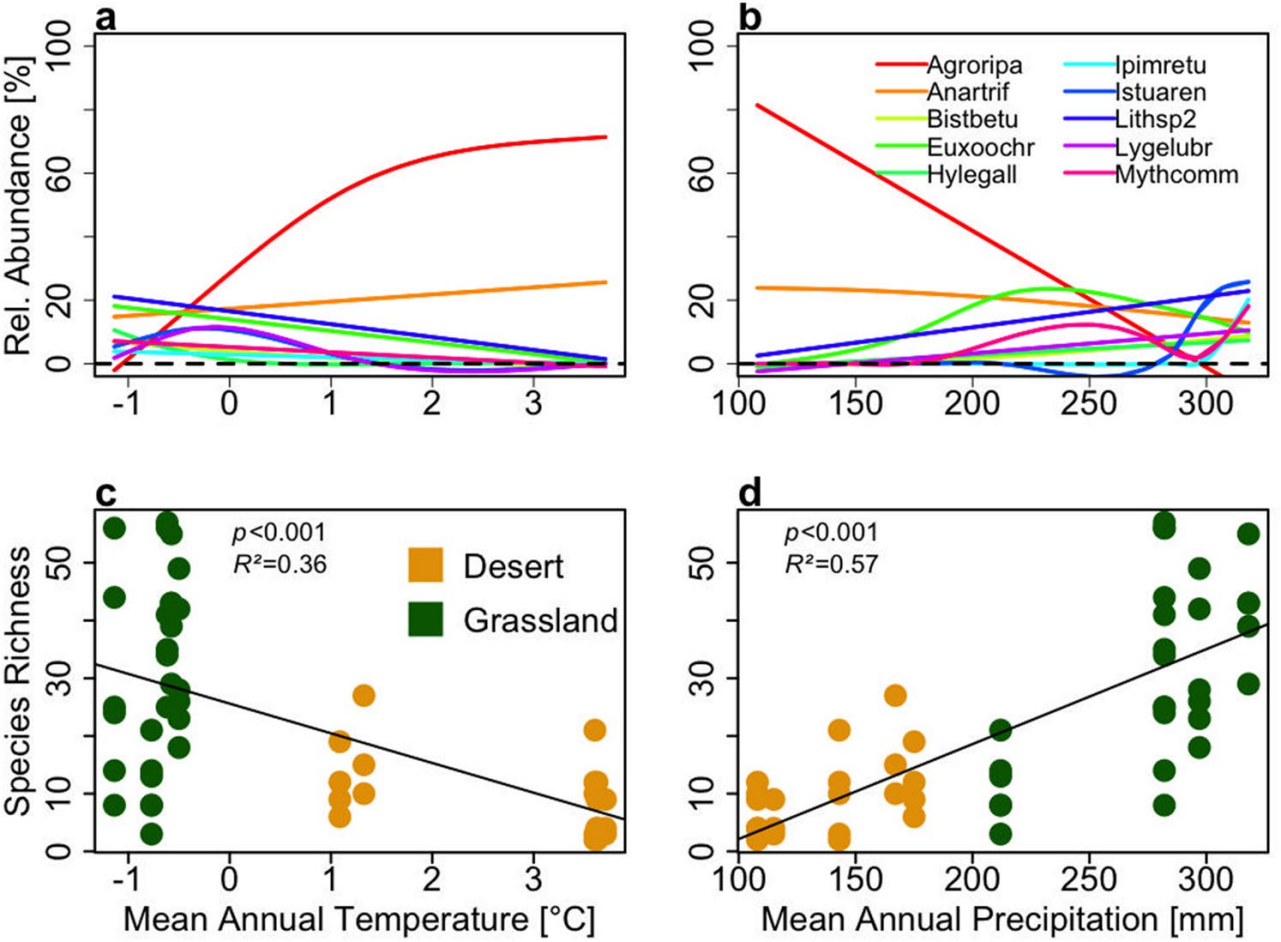
Species relative abundance and species richness impacted by environmental variables. X axes titles are printed only for the lower graphs. GAM shows (**a**) the relative abundance vs. mean annual temperature and (**b**) the relative abundance vs. mean annual precipitation. The general linear regression model demonstrates that moth species richness is impacted by (**c**) mean annual temperature and (**d**) mean annual precipitation. Species abbreviations: *Agrotis ripae* (Agroripa), *Anarta trifolii* (Anartrif), *Biston betularia* (Bistbetu), *Euxoa ochrogaster* (Euxoochr), *Hyles gallii* (Hylegall), *Ipimorpha retusa* (Ipimretu) *Isturgia arenacearia* (Istuaren), *Lithostege sp2* (Lithsp2), *Lygephila lubrica* (Lygelubr), *Mythimna comma* (Mythcomm).

The ten most abundant species responded differently to annual temperature and annual precipitation. *Agrotis ripae* and *Anarta trifolii* showed a decelerating exponential response to increasing annual temperature (Fig. S3 in the appendix), whereas the abundance of *Lithostege* sp. 2 was increasing with increasing annual precipitation. *Hyles gallii, Lygephila lubrica* and *Isturga arenacaria* were mainly present at the more humid northern sites. At low temperature and high precipitation all ten species coexisted, whereas at high temperature and low precipitation, only two species (*Agrotis ripae, Anarta trifolii*) formed the community alone (Fig. 3a,b). A linear regression model shows that species richness of moths was decreasing with increasing annual temperature (*R*^2^ = 0.36, *p* < 0.001) and increasing with rising annual precipitation (*R*^2^ = 0.57, *p* < 0.001). In the grassland sites, species richness was higher than in the desert sites (Kruskal–Wallis Test: *p* < 0.001) (Fig. 3c,d).

We found 96 unique species in total, i.e., species which occurred only in one site. Overall, the unique species numbers of the grassland sites (n = 70) were higher than those of the desert sites (n = 26, Kruskal–Wallis Test: *p* < 0.005). Site 3 had only one unique species, whereas Site 10 had 27 unique species (see Fig. S4 in supplementary material).

### Beta diversity

**Figure 4:**
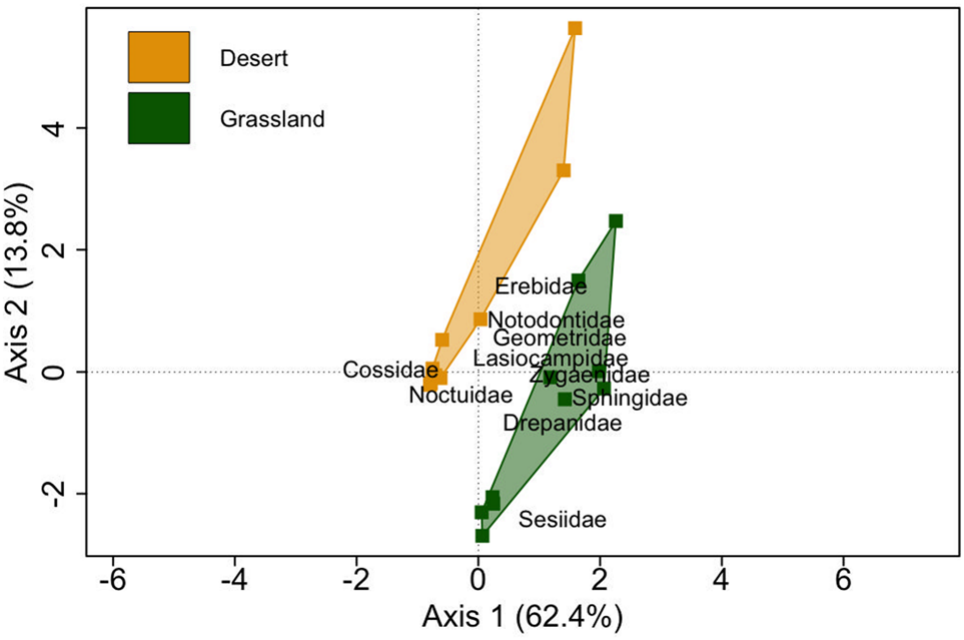
Correspondence analysis of the major families sampled from all sites separated markedly desert (yellow polygon) and grassland (green polygon) sites. Noctuidae and Cossidae were more associated with desert sites, whereas other families were associated with grassland sites. First two axes of the graph together explain 76.2% of the variation.

According to K-means clustering we classified the ten sites into two groups and performed a correspondence analysis based on the family matrix, which indicated clear distinction in the composition of major families in two groups (Fig. 4). Noctuidae and Cossidae were more abundant in the desert sites, while other families were remarkably abundant in the grassland sites. Distinction between these groups was significant (Permanova: *R*^2^ = 0.37, p < 0.006).

Venn diagrams show the species overlap between the moth composition of the desert and the grassland sites in four family groups. The highest overlap was in Noctuids, followed by Erebids and others, the lowest overlap was in Geometrids (see Fig. S5 in supplementary material).

**Figure 5:**
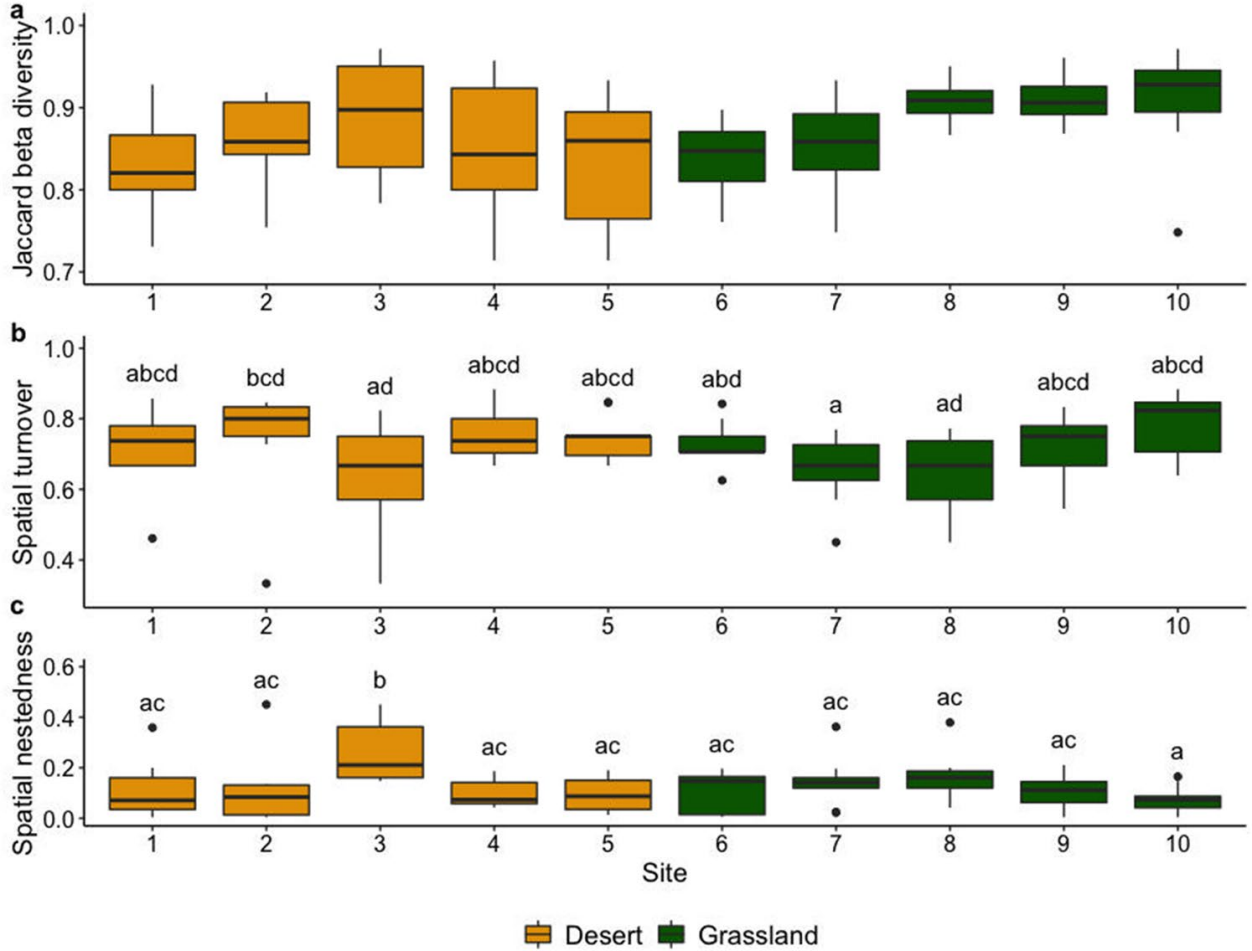
Mean pair-wise (**a**) Jaccard beta diversity, (**b**) spatial turnover, (**c**) spatial nestedness of the ten study sites. Diversity metrics were compared with Wilcoxon test based on the average diversity measures of each site. Different letters show significant differences between sites.

Mean beta diversity of macro-moth species among the sites as calculated with Jaccard’s index was intermediate; β_j_ = 0.82 (range = 0.78–0.86). The outer sites of the gradient with the more extreme environmental conditions had the highest average beta diversity, while sites in the middle had the lowest average beta diversity (Fig. 5a). A linear regression model indicated that with increasing distance Jaccard’s similarity decreases (*R*^2^ = 0.52 *p* < 0.001) (see Fig. S6 in supplementary material).

We checked the contributions of spatial turnover and nestedness to the result of mean beta diversity. Taken together, the contribution of spatial turnover (mean β_t_ = 0.69; range = 0.60–0.77) was much higher than that of nestedness (mean β_t_ = 0.13; range = 0.08–0.27), which means that species replacement was higher than species loss or gain.

Regarding pair-wise beta diversity, Sites 2 and 10 were significantly higher than other sites in terms of turnover (Fig. 5b). Only Site 3 was significantly higher in terms of nestedness (Fig. 5c), all other sites, except Site 8 were not significantly different. The sites with the highest and lowest average species replacement were the same as those with the highest and lowest beta diversity (Fig. 5a).

**Figure 6:**
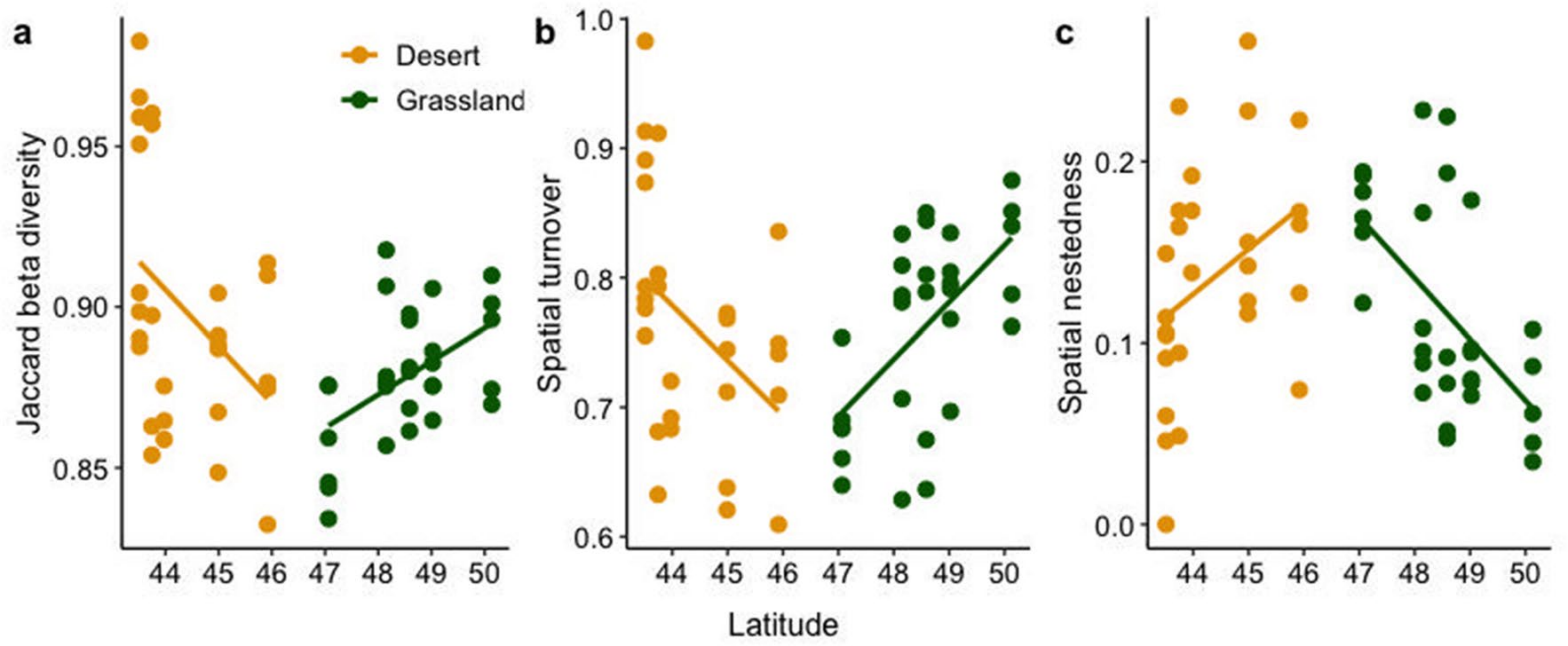
Beta diversity measures along the latitudinal gradient: (**a**) Jaccard’s beta diversity along latitude, (**b**) spatial turnover along latitudinal gradient and (**c**) spatial nestedness along latitude. Desert sites (1–5), grassland sites (6–10).

We found a breakpoint at 46°N as a result of the piecewise regression of Jaccard’s beta diversity, spatial turnover, and spatial nestedness versus latitude (Fig. 6). The fit of the piecewise regression models was significantly higher than the simple linear regression models for all components: *R*^2^ increased from 0.02 to 0.16 (Anova: *F*_2, 52_ = 5.26, *p* < 0.001) for Jaccard beta diversity, from 0.01 to 0.19 (Anova: *F*_2, 52_ = 7.36, *p* < 0.001) for spatial turnover, and from 0.05 to 0.26 (Anova: *F*_2, 52_ = 8.50, *p* < 0.001) for spatial nestedness.

Jaccard’s beta diversity indices significantly differed above (slope = 0.004) and below 46°N (slope = − 0.09) and showed an opposing trend (*R*^2^ = 0.16 *p* < 0.001). Moreover, spatial turnover and nestedness responded in opposite directions with latitude and were significantly different in the desert and the grassland biomes. In the desert, species turnover showed a decreasing trend (slope = − 0.54) (*R*^2^ = 0.19, *p* < 0.001); in contrast, nestedness showed an increasing trend (slope = 0.45) (*R*^2^ = 0.26, *p* < 0.001). In the grassland, species turnover (slope = 0.03) and nestedness (slope = − 0.02) showed contrasting trends. In the desert sites, moth communities’ species loss or gain was dominant, while for the grassland sites, species replacement played the dominant role. The breakpoint of beta diversity pattern was matched by an RDA analysis of plant communities (see Fig. S7 in supplementary material). We found also a breakpoint at 46° N as a result of the piecewise regression of precipitation (*R*^2^ = 0.96, p < 0.001) and temperature (*R*^2^ = 0.89, p < 0.001).

The next step was to test the correlation between environmental variables and beta diversity components, and environmental variables were affecting spatial turnover and spatial nestedness differently. *Precipitation* and *vegetation cover* were positively correlated with turnover, whereas negatively correlated with nestedness. In contrast, *temperature*, *livestock number*, and *wind* were negatively correlated with turnover, while positively correlated with nestedness (Table 2). In the desert, none of the environmental variables was significant for nestedness.

**Table 2:** Pearson correlation coefficients between environmental variables and beta diversity measurements for total (along whole latitudinal gradient), above (> 46°) and below (< 46°) the 46° of latitude.

		Turnover	Nestedness
Precipitation	Total	0.22	− 0.34**
	> 46°	0.60***	− 0.56**
	< 46°	0.18	− 0.19
Temperature	Total	− 0.006	0.14
	> 46°	0.22	− 0.27
	< 46°	0.09	− 0.06
Vegetation cover	Total	0.41**	− 0.45***
	> 46°	0.55**	− 0.49**
	< 46°	0.36*	− 0.34
Livestock number	Total	− 0.40**	0.41**
	> 46°	− 0.54**	0.50**
	< 46°	− 0.23	0.22
Wind	Total	− 0.11	0.25
	> 46°	− 0.56**	0.52**
	< 46°	0.40*	− 0.37
Altitude	Total	− 0.05	0.18
	> 46°	− 0.57**	0.57**
	< 46°	0.37	− 0.36

Significant variables are shown in bold with stars indicating the level of significance.

Using procrustes analysis we compared the distance matrix of moth species with distance matrices of *vegetation* and *livestock*. The matrix of moth species was highly significantly correlated with both matrices of *vegetation* (*r* = 0.74, *P* = 0.001) and *livestock number* (*r* = 0.80, *P* = 0.002), thus corroborating their strong influence on moth community patterns.

In addition, we analyzed if there existed an interaction between environmental variables and biome types and the species richness and diversity of macro-moths (see the results of the negative binomial generalized regression and linear regression in Table S8 in the supplementary material). Interaction effects were only found for the *livestock*, *wind*, and *elevation*. Depending on the biome type species richness of macro-moths responded to *livestock*, *wind*, and *elevation* differently. In the grassland these factors affected the species richness of moths negatively, whereas in the desert there was no effect (Fig. S8).

## Discussion

We studied alpha and beta diversity of macro-moths and associated environmental variables along a large-scale latitudinal gradient in Mongolia for the first time. Against our expectation, we detected two distinct moth communities along the latitudinal gradient, which significantly changed between Site 5 (Dundgobi Aimag) and Site 6 (Tuv Aimag) at 46° N. We assume that this distinction is driven by the pronounced climatic gradient, namely *precipitation* and *temperature*. In piecewise regression of diversity on the *precipitation* and *temperature* we demonstrated this split at 46° N. As we hypothesized, we observed higher moth species richness and species diversity in the grassland sites than in the desert sites. In contrast, moth abundance was lower at grassland sites than in the desert sites. This contradicts with a study on darkling beetles in Mongolia in which species richness declined gradually with latitude. This contrast between moths and beetles could be explained by a higher temperature and desiccation tolerance observed in beetles’^45^. Our study results were in line with the study of Ahlborn et al.^27^, who studied plant communities. In both studies, species richness was low in Site 3 (Tsogtovoo Soum, Khetsuu khoshuu) indicating the need for extra conservation for these transitional sites. In terms of the moth population, our observation of higher species richness and lower abundance in the grassland could be explained by the theory of competitive exclusion. There is higher plant heterogeneity in the grassland, which could ultimately reduce competitive exclusion in the moth population, allowing for the maintenance of several species (high richness) at a similar proportion (similar abundance across species/high evenness). In contrast, lower species richness and higher abundance of certain tolerant species adapted to the few plant species growing in desert prevail^2^.

The differentiating species richness and species diversity of moths between the desert and the grassland sites could be explained by the biotic (*plant species richness* and *livestock number*) and abiotic (*precipitation* and *wind*) variables, which were significantly correlated with the diversity of moths as measured by Hill numbers. Since herbivorous insects rely on plants, both in larval and adult stages, as their food and habitat, it is logical to expect a higher moth species richness in areas with a higher plant species richness^46^. Indeed, variable *Forb* was highly positively correlated with the Hill numbers. In contrast, variable *Goat* was the significant factor among all livestock types and negatively correlated with moth species richness. Herders raise high numbers of goats for income from cashmere, especially in Gobi desert, as one of the common export products of Mongolia^35^. Water and energy (i.e. temperature) availability are the important factors determining overall species richness along the latitudinal gradient. *Precipitation* is the limiting factor for species diversity in the south, while *temperature* is the limiting factor in the north in several taxa^24,25^. In our study, only *precipitation* was a significant variable, positively correlated with the Hill numbers.

While variable *Wind* was negatively correlated with the Hill numbers and similar patterns were observed in other studies related to wind on moth catches^47^. In the first year of the sampling period, strong wind negatively affected the southern sites’ catch successes.

In a study of moths in Finland^48^ the authors observed a contrasting pattern with species that were expanding their ranges poleward due to global warming and were increasing in species richness and decreasing in abundance over time in higher latitudes. The higher abundances in the desert sites in our case were, however, due to only the two heavily dominant Noctuidae species, namely *Agrotis ripae* and *Anarta trifolii*.

At almost all sites, *Agrotis ripae* and *Anarta trifolii* occurred; they were the most abundant species. *A. ripae,* which is called “sand dart moth”, lives mainly in sand dune areas; the caterpillars rest in the sand during the daytime and come out to feed at night^49^. Habitats are characterized by bare ground with sparse vegetation. The study of Spalding et al.^50^ showed that bare ground is an essential factor for the sand dune moth species, such as *Luperina nickerlii;* disturbance could be helpful to create bare ground. Due to desertification and livestock trampling, the soil becomes more sandy and loose; this will create more suitable living conditions for *A. ripae*. Both *A. ripae* and *A. trifolii* can be regarded as generalists and highly migrant species. Their mobility increases with temperature^51^. Thus, both species appear to be suitable indicators of global warming and desertification.

In the grassland sites, the number of unique species was higher than in the desert sites, which implies that in suitable habitats, like grassland sites, more specialists occurred that were adapted to specific habitats. In contrast, in harsher, more arid habitats like desert sites, more generalists occurred. Rabl et al.^46^ found only a small number of unique species in a relatively species-poor rainforest area (i.e., in a creek habitat). Similarly, Beitzholtz and Franzen^51^ reported that specialists prefer suitable habitats; they are prone to stick to their habitats and vulnerable to extinction. Species, such as generalists, are even benefiting disturbance, while specialists are declining^1,4^. Moreover, the number of generalists and specialists are related to beta diversity. Beta diversity increases as the number of specialists increase^52,53^.

Moth species’ host plant preferences could explain differences in major family composition in the desert and the grassland sites. In the desert sites, the moth assemblage composition mainly consists of Noctuids and Cossids, while proportions of Geometrids, Erebids, and others were low. In contrast, family ratios were almost the same in all grassland sites. Many Noctuids are not restricted to specific habitats and are generalists (or even cosmopolitans) in comparison to members of other families (Common 1990). For example, *A. ripae* is polyphagous^54^ and usually, polyphagous species can better survive in disturbed areas.

Most adults of Sphingidae, Geometridae, and Arctiinae usually feed on flower nectar, while most caterpillars of Notodontidae, Drepanidae, and Lasiocampidae mostly feed on the leaves of trees and shrubs^49^. Several species whose larvae feed on trees and undergrowth were found in Sphingidae, Geometridae, and Arctiinae in the grassland sites; thus, we suggest that surrounding forest and shrubs were also responsible for the higher species richness of these families in the grassland sites. In addition, the species richness of Arctiinae is high in areas with complex vegetation types^55^. This can explain the high richness of Erebidae in the grassland sites. Venn diagrams (Figure S5) also showed that species overlap between the desert and the grassland sites of Noctuids, Erebids, and others were similar in percentage (20–27%); in contrast, the species overlap of Geometrids was very low with only four species in common (7.4%). Geometrid moths are sensitive to the environmental changes; thus, the low overlap of Geometrids could indicate better habitat quality in the grassland sites compared to the desert sites.

Beta diversity was mainly driven by species replacement rather than species nestedness. Average pair-wise beta diversity and spatial turnover were high in the external sites and gradually decreased towards the middle of the gradient; in contrast, average nestedness was high in the middle and low in the outer parts. The macro-moth assemblages at northern and southern sites were shaped by forest-steppe and desert, habitats that are distinct from each other. Habitat differences gradually decrease to the middle part, where the steppe runs in gently undulating terrain and becomes a transition zone between these habitats resulting in less difference among moth assemblages. The higher beta-diversity in the outer parts results from high species turnover, while nestedness or difference in species numbers played a less critical role. A similar diversity pattern was reported by Paknia et al.^45^ in Mongolian tenebrionid beetle communities. Generally, turnover is due to abiotic factors, while nested patterns may be attributed to species loss caused by high *livestock numbers* and low *precipitation*.

Intensive land use transforms habitats, making them more similar. The more similar habitats become, the less diverse species they can support. Relative to the larger pool of species found across more distinct habitats, this more homogeneous subset of species becomes capable of dispersing further in more homogeneous habitats. In addition to enhanced dispersal capabilities, more homogenous habitats can support more generalist species that have broad niches. Overall, such traits can decrease beta diversity. However, there is a nuanced caveat. Due to the homogeneity of the habitat, a few tolerant species may persist, leading to species loss which can result in higher beta diversity due to nestedness^37^. In comparison, we observed species replacement happened in areas with high precipitation and high vegetation cover which increases the beta diversity.

Average beta diversity along the latitudinal gradient had a breakpoint, which was revealed at 46° N, indicating a change in moth communities between desert and grassland sites. In arid areas south of 46° N, turnover decreased, and nestedness increased. In contrast, in wet areas north of 46° N, turnover increased, and nestedness decreased. In arid areas species richness decreased, and beta diversity was due to species loss, indicating lower productivity within a harsh environment. The decreasing turnover in the southern sites thus mirrors the physical limiting factor (i.e., lower precipitation). This contrasting patterns of turnover and nestedness have been documented in several studies^23,52^.

A breakpoint in both precipitation (mean annual precipitation: 193 mm) and temperature (mean annual temperature: 0.15 °C) was also found at 46° N. Since the breakpoints are overlapping, we predict that as global temperatures continue to rise, the grassland sites will become more similar to desert sites. In turn, we predict that this trend towards habitat homogenization will lead to a more nested pattern of moth diversity.

Temperature had no significant effect on beta diversity patterns of moths along the latitudinal gradient, both above and below 46°N. Higher precipitation rate, and higher vegetation cover and diversity were responsible for the higher beta diversity in northern sites. *Precipitation* was also a significant variable for species richness.

The results of Procrustes analysis showed that vegetation structure and livestock composition determined the moth assemblage pattern. Along the whole gradient, the effects of *precipitation*, *vegetation cover*, and *vegetation richness* on the species richness and diversity of macro-moths did not change regardless of biome type. However, *livestock*, *altitude* and *wind* affected the species richness and diversity of moths differently, depending on the biome type. In the desert, the vegetation is scarce even without livestock grazing, and the climatic effect is stronger than the effect of livestock grazing. The dynamic equilibrium model could explain the insensitivity of macro-moths of the desert to the number of livestock. In the arid environment, the impact of precipitation overrides the influence of disturbance (in our case, livestock grazing)^56^. In the desert, decreasing species richness and diversity of moths with increasing *altitude* and *wind speed* can be attributed to their low ranges of thermal tolerance compared to the moths in the grassland^57^. Thus, moths living in higher altitude arid environments are in more danger of becoming extinct due to global warming.

Our study shows how moth diversity changes in Central Asia from south to north over a long latitudinal transect and assesses the environmental factors responsible for those changes. Identifying the community composition pattern is useful for the conservation of not only moths, but also biodiversity in general. Our species list represents 51% of all estimated moth species along the latitudinal gradient in Mongolia; this result is the most up to date and systematically collected baseline data for future research.

Moths of the desert Site 3 were more vulnerable to a decrease in species diversity because of low precipitation and high livestock numbers. The local reduction of alpha diversity may result in reduced gamma diversity on regional level. Since 1940, the temperature in the area has increased by 2 °C, while *precipitation* has decreased by 7%. At the same time the number of *goats* increased from four million to 20 million, and large-scale fires occurred repeatedly. As a result, the desert in the south is expanding more and more to the northern part of Mongolia^58^. The most negative effect of livestock is due to the high number of goats. Although cashmere from goats is one of the main export products of Mongolia, the government should stop its support of this unsustainable agricultural practice. Doing so could at least slow down the future consequences of climate change^37^.

Moths are effective bioindicators^22^. Their contrasting patterns of spatial turnover and nestedness in desert and grassland habitats imply that different conservation approaches are needed. Therefore, we suggest that the whole gradient of the grassland has conservation value. Decreasing the number of goats can improve the situation of pasture overall. However, local diversity patterns could scale up to regional; therefore, we recommend abandoning this transitional zone from grazing for recovery. In addition, Site 1 that exists at the highest elevation can function as a refuge area for biodiversity as mirrored by moths should deserve conservation management by excluding livestock grazing.

In contrast, the species richness of the desert sites is similar except for Site 3 (species poor site) and one (species rich site). Thus, there is no exceptional management required for desert Sites 2, 4, and 5.

The high abundances of *A. ripae* and *A. trifolii* indicate that the process of desertification has already intensified and even at those sites some specialists could have already been extirpated before our study. In the future, we aim to study the co-effect of climatic variables and livestock grazing on moth communities at different latitudes. Specifically, we will aim to investigate whether *A. ripae* and *A. trifolii* are indicators of grazing. In addition, we aim to reveal latitude level indicator species, which could be used as reference species to study the migration of moths due to climate change.

## Methods

### Study area

Our study was conducted in the provinces of Umnugobi Aimag, Dundgobi Aimag, Tuv Aimag and Selenge Aimag in Mongolia, at ten study sites located along the latitudinal gradient from the Gobi Desert in the south to the Siberian forests in the north, covering various climatic zones^36^. The southernmost site (43° N, 104° E) is located in semidesert (annual precipitation 146 mm, mean annual temperature − 3.45 °C), while the northernmost site (50° N, 105° E) is located in forest steppe (annual precipitation 318 mm, mean annual temperature − 0.56 °C) (Fig. 1). Livestock herding is one of the major economic sectors in Mongolia, with > 65 million animals^36^. Detailed information on the study sites is given in supplementary material Table S1. We followed the study design of Lang et al.^59^ and Ahlborn et al.^27^ and sampled seven of their original 15 study sites that were spread at a south–north gradient of 600 km. We added three further sites to this transect in northern direction, totaling in a transect length of 860 km.

### Moth sampling

Moths were attracted with recently developed LED lamps (“LepiLED”, height ca. 88 mm, diameter ca. 62 mm, with four UV LEDs (365 nm), two blue (450 nm), one green (530 nm) and one cool white LED)^60^ in combination with Bioform light “towers” (large R. Müller light trapping tower, mesh size 1 mm, 70 cm diameter, 180 cm high) and EasyAcc 26 Ah power bank batteries. For moth collection, killing jars filled with CN were used. All samples were sorted to morphospecies level in the field and kept in glassine envelopes. Moths were sampled manually because the method usually better covers small species than automatic traps^61^. Sampling took place from 9.00 to 12.00 p.m. To avoid temporal effects, specimens were collected in two consecutive years in 2018 (June–July) and in 2019 (July–August) at the peak of vegetation season leaving out nights dominated by full moon. This period covers the flight season of most nocturnal moth species in Mongolia^22^. At each site and in each year, we sampled with three replicates (ten sites × two years × three nights = 60 sampling nights). The southern five sites are located in desert and xeric shrublands biome (desert), and the northern five sites are located in temperate grasslands, savannas & shrublands biome (grassland).

Due to adverse weather conditions five catching nights were successful at some sites (Sites 1, 5, and 10). For analyses, all night samples of each site were aggregated. We brought all samples to Germany and mounted and identified specimens using identification keys^49^ and online identification web sites for moths and butterflies^62,63^. Afterwards, we submitted one or two specimens of each morphospecies for DNA barcoding to Canadian Centre for DNA Barcoding (CCDB) to corroborate our identification of morphospecies. The results on the creation of a DNA barcode library for the collected species will be published in a separate paper (in preparation). Superfamilies of Mimallonoidea, Drepanoidea, Lasiocampoidea, Bombycoidea, Geometroidea, and Noctuioidea are included in the clade of macroheterocera^64^. In this study we also included Sesiidae, Zygaenidae and Cossidae because of their traditional assignment to the (non-monophyletic) macro-moths.

### Environmental data

We included *precipitation, temperature, wind, altitude, plant cover, plant species composition* and the *number of livestock* as environmental variables. We obtained climatic variables from WorldClim dataset^65^. To study vegetation structure, we measured *vegetation cover* and plant species richness in a 10 m × 10 m area with five replications per site. Livestock droppings were counted in the plots to assess grazing pressure. We received vegetation data from Julian Ahlborn (Leibniz Centre for Agricultural Landscape Research) and Christine Römermann (University of Jena) for comparison and easier identification of our samples in the field. Botanist Tungalag Radnaakhand (National University of Mongolia) verified the identification of plant species from dried specimens of our herbarium. We obtained livestock abundance data for each site from the National Statistical Office of Mongolia^66^ (Table S6). We measured coordinates and elevation of the sites with a Garmin Oregon 700 GPS.

### Data analysis

Prior to analyses, we checked all variables for normal distribution by using QQ plot. Depending on these results we chose the appropriate statistical tests or applied log-transformation to normalize data for calculation.

### Alpha diversity

We quantified moth alpha diversity (Hill numbers) of each site, i.e., species richness (q = 0), Shannon diversity, the exponential of Shannon entropy (q = 1), and the reciprocal Simpson’s diversity (q = 2) using the R-package ‘vegan’^67^. We estimated species richness with iChao1 index using R-package *SpadeR*. This index is an improved version of Chao1. To estimate species richness, it uses rare species or the number of singletons. To compare species richness, species diversity, and abundances of all macro-moths of each site along the latitudinal gradient and explore the community pattern at the species and family levels, we used the non-parametric Wilcoxon tests based on data from sampling nights. For comparison the number of unique species of desert and grassland, we used the non-parametric Kruskal–Wallis Test. To study how species richness changes along the climatic gradient, we applied two widely used climatic variables from WorldClim dataset^65^: mean annual temperature (Bio1) and mean annual precipitation (Bio12). We determined niche structure of moth communities along the climatic gradient by analyzing coenoclines of the ten most abundant species. We applied generalized additive models (GAM) with Gaussian distribution and link function to produce the coenoclines. For coenoclines, we used the method of Hoffmann et al.^32^. A general linear model (GLM) was used to calculate the relationship between species richness and climatic variables. Pearson correlation was applied to correlate the Hill numbers of each site with environmental variables.

### Beta diversity

To investigate the major family composition of communities we performed correspondence analysis using the R-package ‘vegan’^28^. K-means clustering of unsupervised learning algorithm was applied to ten sites to cluster them into groups based on their similarity. Clustering was conducted on major family matrices with Hellinger transformation. To study species composition differences between macro-moth communities, we applied permanova on species composition matrix (log + 1 transformation with Bray–Curtis similarity) using *adonis* function of the R-package ‘vegan’. To visualize species overlap between desert and grassland sites, we draw Venn diagrams by using the ‘ggvenn’ package^68^. Southern sites in desert biome are shown in yellow, northern sites in grassland biome are shown in green.

For calculating the pairwise beta diversity among sites and also species composition differences along the latitudinal gradient, we applied the Baselga’s^33^ approach with Jaccard’s dissimilarity index, which partitions beta diversity into two components: spatial turnover and nestedness^34,52^. Partitioning beta diversity measurements are essential to understand the differences between communities; even if two sites have the same beta diversity, the difference can be due to species replacement or species loss or gain^23^.

Spatial turnover is the replacement of some species by other species from one site to the next. Nestedness implies that the species assemblage of a species-poor site is the subset of a different species-rich site. We used the R package ‘betapart’^69^ to calculate beta diversity and its respective partitions. Sampling nights with only one species were excluded from the analysis. We used non-parametric Wilcoxon tests to compare Jaccard’s beta diversity, spatial turnover and nestedness among sites based on data from sampling nights. Piecewise regressions were used to reveal a breakpoint of beta diversity between macro-moth communities along the latitudinal gradient. We examined breakpoints between 43° and 50° with a 1° interval and chose a breakpoint with the lowest residual standard error^70^. We performed this procedure for the beta diversity components separately. We compared piecewise regression models with corresponding simple linear regression models with ANOVA to estimate the improvement of the model fit. To check the model fit, we also compared the R^2^ of piecewise regression models with the R^2^ of the simple linear regression models.

We used Procrustes analysis in R package ‘vegan’ to compare the distance matrix of the moth community with distance matrices of the vegetation guild and livestock abundances at the sites. A significant result demonstrates the similarity of a matrix with a target matrix suggesting an interaction of the observed patterns. To study how the interaction between biome type and environmental variables affect the species richness and the diversity of macro-moths across the whole gradient, we applied generalized linear regression model with negative binomial family and linear regression, respectively. Negative binomial distribution is applied to avoid overdispersion. To fit the negative binomial generalized model, we used glm.nb function of ‘MASS’ package and to fit the linear regression lm function of ‘stats’ package were used. Precipitation, vegetation cover, vegetation richness, livestock, wind, and altitude were included in the model as a predictor variable, while species richness, Shannon diversity, Simpson diversity were response variables. For additive and interaction models, biome was used as a categorical variable. For each predictor variable we built three models: (1) using only a predictor variable without biome, (2) additive model: predictor variable + biome, (3) interaction effect: predictor variable × biome. For choosing the best model between these three models for each predictor variable, we used Akaike’s Information Criterion (AIC).

All analyses were performed using R version 3.6.3^71^.

## Supplementary Information

Supplementary Information.

## Data Availability

Species list of all sites and other supporting information can be found in the Supplementary Material of this article.
