# Supplementary material for: Alpha and beta diversity patterns of macro-moths reveal a breakpoint along a latitudinal gradient in Mongolia
Source: Sci Rep. 2021 Jul 22;11:15018. doi: 10.1038/s41598-021-94471-3 (PMC8298579; doi:10.1038/s41598-021-94471-3)
Supplement: Supplementary file 1 — Supplementary Information. [file 41598_2021_94471_MOESM1_ESM.docx]

**Supplementary material**

**Alpha and beta diversity patterns of macro-moths reveal a breakpoint along a latitudinal gradient in Mongolia**

Khishigdelger Enkhtur ^1,^ *, Gunnar Brehm^2^, Bazartseren Boldgiv ^3, 4^ and Martin Pfeiffer ^1^

^1^Department of Biogeography, University of Bayreuth, Universitätsstraße 30, 95447 Bayreuth, Germany; [khishigdelger.enkhtur@uni-bayreuth.de](mailto:khishigdelger.enkhtur@uni-bayreuth.de); [martin.pfeiffer@uni-bayreuth.de](mailto:martin.pfeiffer@uni-bayreuth.de)

^2^Phyletisches Museum, Institut für Zoologie und Evolutionsbiologie, Friedrich‐Schiller‐Universität, Vor dem Neutor 1, 07743 Jena, Germany; [gunnar.brehm@uni-jena.de](mailto:gunnar.brehm@uni-jena.de)

^3^Ecology Group, Department of Biology, National University of Mongolia, Ikh Surguuliin Gudamj 1, Ulaanbaatar 14201, Mongolia, National University of Mongolia, Ulaanbaatar, Mongolia; [boldgiv@num.edu.mn](mailto:boldgiv@num.edu.mn)

^4^Academy of Natural Sciences of Drexel University, Philadelphia, PA 19103, USA, Academy of Natural Sciences of Drexel University, Philadelphia, United States of America

*****Correspondence: [khishigdelger.enkhtur@uni-bayreuth.de](mailto:khishigdelger.enkhtur@uni-bayreuth.de); Tel.: +49 1575 3063 018

Table S1 Detailed description of study sites

| **Site** | **Group** | **Site names** | **Coordinates** | **Altitude** | **Sampling period** |
| --- | --- | --- | --- | --- | --- |
| 1 | Desert | Umnubobi Aimag, Bayandalai Soum, Khuv, Khaalganii khuudas | 43.74442  103.59985 | 2163 | June 2018, August 2019 |
| 2 | Desert | Umnugobi Aimag, Dalanzadgad, Shatiin am | 43.51139  104.23272 | 1916 | June 2018, August 2019 |
| 3 | Desert | Umnugobi Aimag, Tsogtovoo Soum, Khetsuu khoshuu | 43.97555  105.1534 | 1542 | June 2018, August 2019 |
| 4 | Desert | Dundgobi Aimag, Khuld Soum, Dov Dev | 44.99908  105.61159 | 1218 | June 2018, August 2019 |
| 5 | Desert | Dundgobi Aimag, Saintsagaan Soum, Khargim | 45.91909  106.29333 | 1471 | June 2018, July 2019 |
| 6 | Grassland | Tuv Aimag, Bayan-Unjuul Soum, Khalzan Ukhaa | 47.06541  106.55341 | 1439 | June 2018, July 2019 |
| 7 | Grassland | Tuv Aimag, Ulaanbaatar city, Songinokhairkhan district, 21st khoroo, Partizan, Tsagaan chuluutiin am | 48.14886  106.73609 | 1283 | June 2018, July 2019 |
| 8 | Grassland | Selenge Aimag, Tunkhel village, Khailaast | 48.5862  106.75533 | 1070 | June 2018, July 2019 |
| 9 | Grassland | Selenge aimag, Baruun kharaa soum, Bayangol | 49.02225  106.10812 | 834 | June 2018, July 2019 |
| 10 | Grassland | Selenge Aimag, Zuunburen Soum, Gangiin tokhoi | 50.13164  105.99805 | 619 | June 2018, July 2019 |

Table S2 Number of captured individuals and species richness in each sampling site based on the collection of 2018 and 2019.

| # | **Family** | **Species** | **1** | **2** | **3** | **4** | **5** | **6** | **7** | **8** | **9** | **10** |
| --- | --- | --- | --- | --- | --- | --- | --- | --- | --- | --- | --- | --- |
| 1 | Cossidae | *Acossus sp* | 0 | 0 | 0 | 1 | 1 | 0 | 0 | 0 | 0 | 0 |
| 2 | Cossidae | *Catopta sp* | 3 | 0 | 0 | 0 | 0 | 0 | 0 | 0 | 0 | 0 |
| 3 | Cossidae | *Cossidae sp1* | 0 | 0 | 0 | 0 | 1 | 0 | 0 | 0 | 0 | 0 |
| 4 | Cossidae | *Cossidae sp2* | 0 | 0 | 0 | 0 | 1 | 0 | 0 | 0 | 0 | 0 |
| 5 | Cossidae | *Cossidae sp5* | 0 | 0 | 0 | 0 | 0 | 1 | 0 | 0 | 0 | 0 |
| 6 | Cossidae | *Eogystia hippophaecolus* | 15 | 0 | 0 | 6 | 4 | 0 | 0 | 0 | 0 | 0 |
| 7 | Cossidae | *Eogystia sibirica* | 0 | 0 | 0 | 1 | 0 | 3 | 0 | 0 | 0 | 0 |
| 8 | Drepanidae | *Drepana falcataria* | 0 | 0 | 0 | 0 | 0 | 0 | 1 | 0 | 0 | 0 |
| 9 | Drepanidae | *Tethea ocularis* | 0 | 0 | 0 | 0 | 0 | 0 | 0 | 1 | 0 | 1 |
| 10 | Drepanidae | *Thyatira batis* | 0 | 0 | 0 | 0 | 0 | 0 | 0 | 0 | 0 | 1 |
| 11 | Erebidae | *Arctia flavia* | 0 | 0 | 0 | 0 | 0 | 0 | 1 | 2 | 0 | 1 |
| 12 | Erebidae | *Arctia matronula* | 0 | 0 | 0 | 0 | 0 | 0 | 0 | 3 | 0 | 0 |
| 13 | Erebidae | *Atolmis rubricollis* | 0 | 0 | 0 | 0 | 0 | 0 | 1 | 1 | 0 | 1 |
| 14 | Erebidae | *Callistege fortalitium* | 0 | 0 | 0 | 0 | 0 | 0 | 0 | 0 | 1 | 0 |
| 15 | Erebidae | *Calyptra thalictri* | 0 | 1 | 0 | 0 | 0 | 0 | 0 | 0 | 4 | 4 |
| 16 | Erebidae | *Catocala bella* | 0 | 0 | 0 | 0 | 0 | 0 | 0 | 0 | 0 | 5 |
| 17 | Erebidae | *Catocala deuteronympha* | 0 | 0 | 0 | 0 | 0 | 0 | 0 | 1 | 0 | 83 |
| 18 | Erebidae | *Catocala fulminea* | 0 | 0 | 0 | 0 | 0 | 0 | 0 | 0 | 0 | 1 |
| 19 | Erebidae | *Catocala nupta japonica* | 0 | 0 | 0 | 0 | 0 | 0 | 0 | 0 | 0 | 31 |
| 20 | Erebidae | *Catocala pacta* | 0 | 0 | 0 | 0 | 0 | 0 | 0 | 0 | 0 | 16 |
| 21 | Erebidae | *Chelis dahurica* | 0 | 0 | 0 | 0 | 0 | 0 | 4 | 44 | 4 | 0 |
| 22 | Erebidae | *Chelis mongolica* | 0 | 0 | 0 | 0 | 2 | 1 | 0 | 0 | 1 | 1 |
| 23 | Erebidae | *Chrysorithrum flavomaculata* | 0 | 0 | 0 | 0 | 0 | 1 | 2 | 6 | 0 | 0 |
| 24 | Erebidae | *Diacrisia purpurata* | 0 | 0 | 0 | 0 | 0 | 0 | 0 | 1 | 0 | 1 |
| 25 | Erebidae | *Diacrisia sannio* | 0 | 0 | 0 | 0 | 0 | 0 | 0 | 0 | 0 | 2 |
| 26 | Erebidae | *Dicallomera angelus* | 0 | 0 | 0 | 2 | 0 | 0 | 0 | 0 | 2 | 0 |
| 27 | Erebidae | *Drasteria chinensis* | 43 | 11 | 2 | 0 | 30 | 0 | 3 | 3 | 0 | 0 |
| 28 | Erebidae | *Drasteria rada* | 2 | 0 | 0 | 0 | 1 | 0 | 0 | 0 | 1 | 0 |
| 29 | Erebidae | *Eilema flavociliata* | 0 | 0 | 0 | 0 | 0 | 0 | 0 | 0 | 0 | 1 |
| 30 | Erebidae | *Emmelia trabealis* | 0 | 0 | 0 | 0 | 0 | 0 | 2 | 0 | 0 | 1 |
| 31 | Erebidae | *Epatolmis caesarea* | 0 | 3 | 0 | 0 | 0 | 0 | 0 | 0 | 0 | 0 |
| 32 | Erebidae | *Eublemma rosea* | 3 | 0 | 0 | 0 | 1 | 1 | 2 | 0 | 0 | 0 |
| 33 | Erebidae | *Eublemma sp* | 0 | 1 | 0 | 0 | 0 | 0 | 0 | 0 | 0 | 0 |
| 34 | Erebidae | *Euproctis similis* | 0 | 0 | 0 | 0 | 0 | 0 | 0 | 1 | 0 | 5 |
| 35 | Erebidae | *Hypena obesalis* | 0 | 2 | 0 | 0 | 0 | 0 | 0 | 0 | 0 | 0 |
| 36 | Erebidae | *Ivela ochropoda* | 0 | 0 | 0 | 0 | 0 | 0 | 0 | 0 | 1 | 3 |
| 37 | Erebidae | *Leucoma candida* | 0 | 0 | 0 | 0 | 0 | 0 | 1 | 2 | 1 | 22 |
| 38 | Erebidae | *Lygephila ludicra* | 0 | 0 | 0 | 0 | 0 | 0 | 2 | 4 | 171 | 32 |
| 39 | Erebidae | *Lymantria dispar* | 0 | 1 | 0 | 0 | 0 | 0 | 0 | 0 | 0 | 17 |
| 40 | Erebidae | *Polypogon tentacularia* | 0 | 0 | 0 | 0 | 0 | 0 | 0 | 1 | 0 | 0 |
| 41 | Erebidae | *Setina irrorella* | 0 | 0 | 0 | 0 | 0 | 1 | 0 | 1 | 1 | 0 |
| 42 | Erebidae | *Spiris bipunctata* | 0 | 0 | 0 | 0 | 0 | 0 | 0 | 1 | 0 | 1 |
| 43 | Erebidae | *Spiris striata* | 0 | 0 | 0 | 0 | 0 | 0 | 0 | 1 | 0 | 0 |
| 44 | Erebidae | *Stigmatophora flava* | 0 | 0 | 0 | 0 | 0 | 0 | 0 | 0 | 2 | 1 |
| 45 | Erebidae | *Stigmatophora micans* | 0 | 0 | 0 | 0 | 0 | 0 | 2 | 2 | 1 | 1 |
| 46 | Geometridae | *Abraxas grossulariata* | 0 | 0 | 0 | 0 | 0 | 0 | 0 | 0 | 0 | 2 |
| 47 | Geometridae | *Alcis sp1* | 0 | 0 | 0 | 0 | 0 | 0 | 0 | 1 | 0 | 0 |
| 48 | Geometridae | *Arichanna melanaria* | 0 | 0 | 0 | 0 | 0 | 0 | 0 | 0 | 0 | 8 |
| 49 | Geometridae | *Aspitates curvaria* | 0 | 1 | 0 | 0 | 0 | 0 | 0 | 0 | 0 | 0 |
| 50 | Geometridae | *Biston betularia* | 0 | 0 | 0 | 0 | 0 | 0 | 40 | 2 | 23 | 57 |
| 51 | Geometridae | *Cabera leptographa* | 0 | 0 | 0 | 0 | 0 | 0 | 0 | 0 | 0 | 1 |
| 52 | Geometridae | *Catarhoe cuculata* | 0 | 0 | 0 | 0 | 0 | 0 | 9 | 6 | 11 | 1 |
| 53 | Geometridae | *Chiasmia clathrata* | 0 | 0 | 0 | 0 | 0 | 0 | 1 | 3 | 4 | 1 |
| 54 | Geometridae | *Deileptenia sp* | 0 | 0 | 0 | 0 | 0 | 1 | 0 | 0 | 0 | 0 |
| 55 | Geometridae | *Digrammia rippertaria* | 0 | 0 | 0 | 0 | 0 | 0 | 1 | 0 | 0 | 0 |
| 56 | Geometridae | *Epirrhoe pupillata* | 0 | 0 | 0 | 0 | 0 | 0 | 2 | 0 | 0 | 0 |
| 57 | Geometridae | *Epirrhoe supergressa* | 0 | 0 | 0 | 0 | 0 | 0 | 2 | 0 | 0 | 2 |
| 58 | Geometridae | *Euphyia unangulata* | 0 | 0 | 0 | 0 | 0 | 0 | 1 | 0 | 2 | 0 |
| 59 | Geometridae | *Eupithecia centaureata* | 0 | 0 | 0 | 0 | 0 | 0 | 0 | 1 | 0 | 0 |
| 60 | Geometridae | *Eupithecia intricata* | 0 | 1 | 0 | 0 | 0 | 0 | 0 | 0 | 0 | 0 |
| 61 | Geometridae | *Eupithecia laricata* | 0 | 0 | 0 | 0 | 0 | 1 | 0 | 0 | 0 | 0 |
| 62 | Geometridae | *Eupithecia subumbrata* | 0 | 0 | 0 | 0 | 0 | 0 | 0 | 3 | 0 | 0 |
| 63 | Geometridae | *Gagitodes sagittata* | 0 | 0 | 0 | 0 | 0 | 0 | 0 | 0 | 1 | 0 |
| 64 | Geometridae | *Geometra rana* | 0 | 0 | 0 | 0 | 0 | 0 | 0 | 5 | 3 | 3 |
| 65 | Geometridae | *Hemistola veneta* | 0 | 0 | 0 | 0 | 0 | 0 | 0 | 17 | 6 | 45 |
| 66 | Geometridae | *Hylaea fasciaria* | 0 | 0 | 0 | 0 | 0 | 0 | 0 | 0 | 0 | 1 |
| 67 | Geometridae | *Hypomecis punctinalis* | 0 | 0 | 0 | 0 | 0 | 0 | 0 | 0 | 1 | 0 |
| 68 | Geometridae | *Hypomecis roboraria* | 0 | 0 | 0 | 0 | 0 | 0 | 1 | 2 | 0 | 0 |
| 69 | Geometridae | *Idaea serpentata* | 0 | 0 | 0 | 0 | 0 | 0 | 0 | 0 | 0 | 1 |
| 70 | Geometridae | *Idaea straminata* | 0 | 0 | 0 | 0 | 0 | 0 | 0 | 0 | 2 | 0 |
| 71 | Geometridae | *Isturgia arenacearia* | 0 | 0 | 0 | 0 | 0 | 0 | 11 | 3 | 95 | 125 |
| 72 | Geometridae | *Isturgia kaszabi* | 0 | 0 | 0 | 0 | 0 | 0 | 0 | 1 | 2 | 0 |
| 73 | Geometridae | *Jankowskia athleta* | 0 | 0 | 0 | 0 | 0 | 0 | 0 | 18 | 1 | 0 |
| 74 | Geometridae | *Juxtephria consentaria* | 0 | 0 | 0 | 0 | 0 | 0 | 0 | 3 | 5 | 3 |
| 75 | Geometridae | *Lampropteryx sp1* | 0 | 0 | 0 | 0 | 0 | 0 | 0 | 1 | 0 | 0 |
| 76 | Geometridae | *Lithostege sp2* | 0 | 9 | 0 | 3 | 0 | 58 | 11 | 163 | 69 | 22 |
| 77 | Geometridae | *Macaria AH01Mg* | 0 | 0 | 0 | 0 | 0 | 0 | 0 | 1 | 1 | 0 |
| 78 | Geometridae | *Macaria alternata* | 0 | 0 | 0 | 0 | 0 | 0 | 0 | 0 | 0 | 1 |
| 79 | Geometridae | *Macaria artesiaria* | 0 | 0 | 0 | 0 | 0 | 0 | 0 | 0 | 0 | 1 |
| 80 | Geometridae | *Macaria sp 2* | 0 | 0 | 0 | 0 | 0 | 0 | 0 | 0 | 1 | 0 |
| 81 | Geometridae | *Macaria sp 3* | 0 | 0 | 0 | 0 | 0 | 0 | 0 | 0 | 1 | 0 |
| 82 | Geometridae | *Macaria wauaria* | 0 | 0 | 0 | 0 | 0 | 0 | 0 | 3 | 14 | 6 |
| 83 | Geometridae | *Megalycinia strictaria* | 1 | 0 | 0 | 0 | 0 | 0 | 7 | 6 | 26 | 0 |
| 84 | Geometridae | *Megaspilates mundataria* | 0 | 0 | 0 | 0 | 0 | 0 | 0 | 10 | 0 | 17 |
| 85 | Geometridae | *Odontopera bidentata* | 0 | 0 | 0 | 0 | 0 | 0 | 3 | 1 | 0 | 0 |
| 86 | Geometridae | *Pelurga comitata* | 0 | 0 | 0 | 0 | 0 | 0 | 0 | 0 | 5 | 1 |
| 87 | Geometridae | *Phibalapteryx virgata* | 0 | 0 | 0 | 0 | 1 | 0 | 0 | 39 | 34 | 19 |
| 88 | Geometridae | *Rheumaptera hastata* | 0 | 0 | 0 | 0 | 0 | 1 | 0 | 0 | 0 | 0 |
| 89 | Geometridae | *Rhodostrophia jacularia* | 1 | 4 | 5 | 2 | 0 | 2 | 0 | 3 | 0 | 0 |
| 90 | Geometridae | *Rhodostrophia vibicaria* | 0 | 0 | 0 | 0 | 0 | 0 | 1 | 8 | 0 | 0 |
| 91 | Geometridae | *Schistostege nubilaria* | 0 | 0 | 0 | 0 | 0 | 0 | 3 | 3 | 2 | 0 |
| 92 | Geometridae | *Scopula albiceraria* | 0 | 0 | 0 | 0 | 0 | 0 | 0 | 1 | 3 | 0 |
| 93 | Geometridae | *Scopula decorota przewalskii* | 0 | 0 | 0 | 0 | 0 | 1 | 1 | 1 | 0 | 17 |
| 94 | Geometridae | *Scopula immorata* | 0 | 0 | 0 | 0 | 0 | 0 | 0 | 1 | 0 | 0 |
| 95 | Geometridae | *Scopula rubiginata* | 0 | 0 | 0 | 0 | 0 | 0 | 0 | 0 | 0 | 31 |
| 96 | Geometridae | *Scopula subpunctaria* | 1 | 0 | 0 | 0 | 0 | 0 | 1 | 1 | 9 | 1 |
| 97 | Geometridae | *Scopula virgulata* | 0 | 0 | 0 | 0 | 0 | 0 | 6 | 10 | 25 | 2 |
| 98 | Geometridae | *Scotopteryx chenopodiata* | 0 | 0 | 0 | 0 | 0 | 0 | 1 | 36 | 3 | 0 |
| 99 | Geometridae | *Spargania luctuata* | 0 | 0 | 0 | 0 | 0 | 0 | 1 | 0 | 0 | 0 |
| 100 | Geometridae | *Stamnodes danilovi* | 0 | 1 | 0 | 0 | 0 | 0 | 0 | 0 | 0 | 0 |
| 101 | Geometridae | *Thalera chlorosaria* | 0 | 0 | 0 | 0 | 0 | 0 | 0 | 0 | 0 | 1 |
| 102 | Geometridae | *Thetidia chlorophyllaria* | 0 | 0 | 0 | 0 | 0 | 0 | 0 | 1 | 2 | 6 |
| 103 | Geometridae | *Thetidia smaragdaria* | 0 | 0 | 0 | 0 | 0 | 0 | 3 | 6 | 1 | 3 |
| 104 | Lasiocampidae | *Dendrolimus superans* | 0 | 0 | 0 | 0 | 0 | 0 | 3 | 0 | 13 | 0 |
| 105 | Lasiocampidae | *Gastropacha populifolia* | 0 | 0 | 0 | 0 | 0 | 0 | 0 | 1 | 0 | 0 |
| 106 | Lasiocampidae | *Gastropacha quercifolia* | 0 | 4 | 0 | 0 | 0 | 0 | 2 | 33 | 5 | 9 |
| 107 | Lasiocampidae | *Malacosoma castrensis* | 0 | 1 | 0 | 1 | 0 | 0 | 0 | 11 | 1 | 0 |
| 108 | Lasiocampidae | *Malacosoma neustria transmongolicum* | 0 | 0 | 0 | 1 | 0 | 0 | 0 | 0 | 0 | 0 |
| 109 | Lasiocampidae | *Phyllodesma ilicifolia* | 0 | 1 | 0 | 0 | 0 | 0 | 0 | 0 | 0 | 0 |
| 110 | Noctuidae | *Abrostola tripartita* | 0 | 0 | 0 | 0 | 0 | 0 | 1 | 1 | 0 | 0 |
| 111 | Noctuidae | *Actebia fennica* | 1 | 0 | 0 | 0 | 0 | 0 | 1 | 12 | 0 | 0 |
| 112 | Noctuidae | *Actebia poecila* | 3 | 1 | 0 | 0 | 4 | 7 | 2 | 0 | 0 | 0 |
| 113 | Noctuidae | *Actebia praecox* | 0 | 0 | 0 | 0 | 0 | 0 | 0 | 0 | 0 | 2 |
| 114 | Noctuidae | *Actebia squalida* | 0 | 0 | 0 | 1 | 6 | 1 | 0 | 0 | 0 | 0 |
| 115 | Noctuidae | *Agrotis clavis* | 0 | 0 | 0 | 0 | 0 | 0 | 13 | 45 | 3 | 2 |
| 116 | Noctuidae | *Agrotis exclamationis* | 0 | 0 | 0 | 0 | 0 | 2 | 11 | 19 | 4 | 4 |
| 117 | Noctuidae | *Agrotis ipsilon* | 0 | 0 | 0 | 1 | 2 | 1 | 0 | 0 | 0 | 0 |
| 118 | Noctuidae | *Agrotis ripae* | 356 | 6 | 1445 | 1714 | 2413 | 20 | 21 | 10 | 1 | 0 |
| 119 | Noctuidae | *Agrotis segetum* | 0 | 0 | 0 | 0 | 5 | 0 | 0 | 0 | 0 | 0 |
| 120 | Noctuidae | *Amphipoea fucosa* | 0 | 0 | 0 | 0 | 0 | 0 | 0 | 0 | 0 | 4 |
| 121 | Noctuidae | *Anaplectoides prasina* | 0 | 0 | 0 | 0 | 0 | 0 | 0 | 1 | 0 | 0 |
| 122 | Noctuidae | *Anarta colletti* | 1 | 0 | 0 | 0 | 0 | 0 | 0 | 0 | 0 | 0 |
| 123 | Noctuidae | *Anarta stigmosa* | 1 | 0 | 0 | 34 | 4 | 0 | 0 | 0 | 0 | 0 |
| 124 | Noctuidae | *Anarta trifolii* | 191 | 48 | 34 | 125 | 169 | 53 | 110 | 8 | 55 | 8 |
| 125 | Noctuidae | *Apamea lateritia* | 28 | 0 | 0 | 0 | 0 | 0 | 2 | 4 | 3 | 5 |
| 126 | Noctuidae | *Apamea maillardi* | 0 | 0 | 0 | 0 | 0 | 0 | 0 | 0 | 2 | 0 |
| 127 | Noctuidae | *Autographa buraetica* | 2 | 0 | 0 | 0 | 0 | 0 | 1 | 8 | 2 | 0 |
| 128 | Noctuidae | *Bryophila orthogramma* | 0 | 0 | 0 | 0 | 0 | 0 | 0 | 0 | 0 | 1 |
| 129 | Noctuidae | *Calamia decipiens* | 1 | 0 | 0 | 0 | 0 | 0 | 0 | 0 | 0 | 0 |
| 130 | Noctuidae | *Caradrina montana* | 21 | 8 | 8 | 0 | 1 | 0 | 0 | 4 | 0 | 0 |
| 131 | Noctuidae | *Caradrina sp2* | 4 | 0 | 15 | 4 | 0 | 0 | 0 | 0 | 0 | 0 |
| 132 | Noctuidae | *Cardepia sociabilis* | 0 | 0 | 0 | 1 | 0 | 0 | 0 | 0 | 0 | 0 |
| 133 | Noctuidae | *Chersotis ocellina* | 0 | 0 | 0 | 0 | 0 | 0 | 1 | 0 | 1 | 0 |
| 134 | Noctuidae | *Chilodes repeteki* | 0 | 0 | 0 | 1 | 0 | 0 | 0 | 0 | 0 | 0 |
| 135 | Noctuidae | *Coenobia rufa* | 1 | 3 | 0 | 0 | 0 | 0 | 0 | 0 | 0 | 0 |
| 136 | Noctuidae | *Conisania arida* | 0 | 0 | 0 | 0 | 0 | 0 | 2 | 4 | 0 | 0 |
| 137 | Noctuidae | *Cosmia pyralina* | 0 | 0 | 0 | 0 | 0 | 0 | 0 | 0 | 0 | 13 |
| 138 | Noctuidae | *Cryptocala chardinyi* | 0 | 0 | 0 | 0 | 0 | 0 | 0 | 1 | 1 | 0 |
| 139 | Noctuidae | *Cucullia absinthii* | 0 | 0 | 0 | 0 | 1 | 0 | 0 | 0 | 0 | 0 |
| 140 | Noctuidae | *Cucullia argentea* | 0 | 0 | 0 | 0 | 0 | 0 | 0 | 1 | 2 | 6 |
| 141 | Noctuidae | *Cucullia artemisiae* | 0 | 0 | 0 | 0 | 0 | 0 | 0 | 1 | 1 | 0 |
| 142 | Noctuidae | *Cucullia biradiata* | 1 | 0 | 0 | 0 | 0 | 0 | 0 | 2 | 0 | 0 |
| 143 | Noctuidae | *Cucullia cineracea* | 0 | 0 | 0 | 0 | 0 | 1 | 0 | 0 | 2 | 2 |
| 144 | Noctuidae | *Cucullia dracunculi* | 0 | 0 | 0 | 0 | 0 | 0 | 0 | 1 | 3 | 1 |
| 145 | Noctuidae | *Cucullia fuchsiana* | 0 | 0 | 0 | 0 | 1 | 0 | 0 | 0 | 1 | 0 |
| 146 | Noctuidae | *Cucullia scopariae* | 0 | 1 | 1 | 0 | 0 | 0 | 1 | 0 | 2 | 21 |
| 147 | Noctuidae | *Cucullia sp2* | 0 | 0 | 0 | 0 | 0 | 0 | 0 | 0 | 1 | 0 |
| 148 | Noctuidae | *Cucullia splendida* | 4 | 7 | 0 | 1 | 8 | 4 | 1 | 0 | 4 | 13 |
| 149 | Noctuidae | *Cucullia umbratica* | 0 | 0 | 0 | 0 | 0 | 0 | 3 | 2 | 2 | 0 |
| 150 | Noctuidae | *Diachrysia stenochrysis* | 0 | 0 | 0 | 0 | 0 | 0 | 0 | 1 | 0 | 0 |
| 151 | Noctuidae | *Dichagyris kaszabi* | 0 | 3 | 0 | 0 | 0 | 0 | 0 | 0 | 0 | 0 |
| 152 | Noctuidae | *Dichagyris musiva* | 0 | 0 | 0 | 0 | 0 | 0 | 0 | 0 | 0 | 1 |
| 153 | Noctuidae | *Dichagyris* sp | 0 | 0 | 1 | 0 | 0 | 0 | 0 | 0 | 0 | 0 |
| 154 | Noctuidae | *Dichagyris vallesiaca* | 15 | 2 | 0 | 0 | 0 | 0 | 0 | 0 | 0 | 0 |
| 155 | Noctuidae | *Enargia paleacea* | 0 | 0 | 0 | 0 | 0 | 0 | 0 | 0 | 2 | 5 |
| 156 | Noctuidae | *Eremohadena* sp | 0 | 0 | 0 | 0 | 1 | 0 | 0 | 0 | 0 | 0 |
| 157 | Noctuidae | *Euchalcia mongolica* | 0 | 0 | 0 | 0 | 0 | 0 | 1 | 2 | 1 | 1 |
| 158 | Noctuidae | *Euchalcia renardi* | 0 | 0 | 0 | 0 | 0 | 0 | 0 | 2 | 0 | 0 |
| 159 | Noctuidae | *Eurois occulta* | 0 | 0 | 0 | 0 | 0 | 0 | 0 | 0 | 1 | 0 |
| 160 | Noctuidae | *Euxoa basigramma* | 1 | 0 | 0 | 0 | 0 | 0 | 0 | 0 | 0 | 0 |
| 161 | Noctuidae | *Euxoa cursoria* | 91 | 2 | 0 | 0 | 1 | 0 | 1 | 0 | 0 | 0 |
| 162 | Noctuidae | *Euxoa decorans* | 5 | 1 | 0 | 0 | 0 | 0 | 0 | 0 | 0 | 0 |
| 163 | Noctuidae | *Euxoa distinguenda* | 1 | 0 | 0 | 0 | 5 | 2 | 6 | 8 | 19 | 25 |
| 164 | Noctuidae | *Euxoa ochrogaster* | 56 | 3 | 4 | 11 | 56 | 64 | 55 | 21 | 129 | 33 |
| 165 | Noctuidae | *Euxoa oranaria* | 1 | 0 | 0 | 0 | 0 | 0 | 0 | 0 | 0 | 0 |
| 166 | Noctuidae | *Euxoa* sp | 1 | 0 | 0 | 0 | 0 | 0 | 0 | 0 | 0 | 0 |
| 167 | Noctuidae | *Euxoa tritici* | 0 | 0 | 0 | 0 | 0 | 2 | 0 | 2 | 1 | 13 |
| 168 | Noctuidae | *Feltia nigrita* | 0 | 0 | 0 | 0 | 0 | 0 | 0 | 1 | 0 | 0 |
| 169 | Noctuidae | *Hada plebeja* | 0 | 0 | 0 | 0 | 0 | 0 | 0 | 7 | 0 | 0 |
| 170 | Noctuidae | *Hadena aberrans* | 0 | 0 | 0 | 0 | 0 | 0 | 0 | 2 | 1 | 0 |
| 171 | Noctuidae | *Hadena corrupta* | 0 | 0 | 0 | 0 | 0 | 0 | 1 | 1 | 0 | 0 |
| 172 | Noctuidae | *Hadena variolata* | 1 | 1 | 0 | 0 | 0 | 1 | 0 | 2 | 0 | 2 |
| 173 | Noctuidae | *Heliothis adaucta* | 0 | 0 | 0 | 0 | 0 | 0 | 0 | 0 | 2 | 0 |
| 174 | Noctuidae | *Heliothis ononis* | 0 | 0 | 0 | 0 | 0 | 0 | 9 | 2 | 3 | 0 |
| 175 | Noctuidae | *Hoplodrina octogenaria* | 0 | 0 | 0 | 0 | 0 | 0 | 0 | 0 | 1 | 3 |
| 176 | Noctuidae | *Hyssia cavernosa* | 0 | 0 | 0 | 0 | 0 | 0 | 1 | 1 | 0 | 2 |
| 177 | Noctuidae | *Ipimorpha retusa* | 0 | 0 | 0 | 0 | 0 | 0 | 0 | 0 | 0 | 98 |
| 178 | Noctuidae | *Lacanobia aliena* | 0 | 0 | 0 | 0 | 0 | 8 | 1 | 8 | 0 | 0 |
| 179 | Noctuidae | *Lacanobia contigua* | 0 | 0 | 0 | 0 | 0 | 0 | 9 | 1 | 6 | 0 |
| 180 | Noctuidae | *Lacanobia thalassina* | 0 | 0 | 0 | 0 | 0 | 0 | 23 | 20 | 8 | 0 |
| 181 | Noctuidae | *Lasionycta imbecilla* | 0 | 0 | 0 | 0 | 0 | 0 | 0 | 4 | 0 | 0 |
| 182 | Noctuidae | *Lasionycta proxima* | 0 | 0 | 0 | 0 | 0 | 0 | 15 | 30 | 1 | 0 |
| 183 | Noctuidae | *Lygephila pastinum* | 0 | 0 | 0 | 0 | 0 | 0 | 0 | 0 | 1 | 0 |
| 184 | Noctuidae | *Lygephila viciae* | 0 | 0 | 0 | 0 | 0 | 0 | 72 | 11 | 1 | 0 |
| 185 | Noctuidae | *Mamestra brassicae* | 0 | 0 | 0 | 4 | 15 | 0 | 0 | 1 | 0 | 0 |
| 186 | Noctuidae | *Mythimna albiradiosa* | 0 | 0 | 0 | 1 | 3 | 0 | 1 | 0 | 0 | 0 |
| 187 | Noctuidae | *Mythimna comma* | 1 | 0 | 0 | 0 | 0 | 16 | 10 | 12 | 11 | 88 |
| 188 | Noctuidae | *Mythimna conigera* | 0 | 0 | 0 | 0 | 0 | 0 | 0 | 0 | 2 | 6 |
| 189 | Noctuidae | *Mythimna opaca* | 0 | 0 | 0 | 0 | 0 | 0 | 0 | 4 | 1 | 0 |
| 190 | Noctuidae | *Mythimna oxygala* | 0 | 0 | 0 | 0 | 0 | 0 | 5 | 1 | 0 | 1 |
| 191 | Noctuidae | *Mythimna pallens* | 0 | 0 | 0 | 0 | 0 | 0 | 8 | 1 | 0 | 1 |
| 192 | Noctuidae | *Mythimna separata* | 0 | 1 | 0 | 0 | 0 | 0 | 0 | 0 | 0 | 0 |
| 193 | Noctuidae | *Mythimna velutina* | 0 | 0 | 0 | 0 | 0 | 0 | 0 | 7 | 33 | 24 |
| 194 | Noctuidae | *Naenia contaminata* | 0 | 0 | 0 | 0 | 0 | 0 | 0 | 0 | 0 | 1 |
| 195 | Noctuidae | *Ochropleura plecta* | 0 | 0 | 0 | 0 | 0 | 0 | 1 | 0 | 0 | 0 |
| 196 | Noctuidae | *Panchrysia dives* | 0 | 0 | 0 | 0 | 0 | 0 | 0 | 1 | 0 | 1 |
| 197 | Noctuidae | *Panchrysia ornata* | 0 | 6 | 0 | 0 | 0 | 0 | 0 | 0 | 0 | 0 |
| 198 | Noctuidae | *Plusia putnami* | 0 | 0 | 0 | 0 | 0 | 0 | 0 | 0 | 0 | 1 |
| 199 | Noctuidae | *Polia altaica* | 0 | 0 | 0 | 0 | 1 | 0 | 6 | 1 | 0 | 0 |
| 200 | Noctuidae | *Polia bombycina* | 0 | 0 | 0 | 0 | 0 | 0 | 1 | 15 | 11 | 30 |
| 201 | Noctuidae | *Polia nebulosa* | 0 | 0 | 0 | 0 | 0 | 0 | 0 | 3 | 3 | 0 |
| 202 | Noctuidae | *Prognorisma albifurca* | 0 | 0 | 0 | 0 | 0 | 0 | 2 | 0 | 0 | 0 |
| 203 | Noctuidae | *Protoschinia scutosa* | 0 | 1 | 1 | 0 | 0 | 0 | 1 | 0 | 0 | 0 |
| 204 | Noctuidae | *Pyrrhia umbra* | 0 | 0 | 0 | 0 | 0 | 0 | 0 | 1 | 0 | 0 |
| 205 | Noctuidae | *red_noc* | 0 | 0 | 0 | 0 | 0 | 0 | 0 | 0 | 1 | 0 |
| 206 | Noctuidae | *Resapamea mammuthus* | 0 | 0 | 0 | 0 | 0 | 0 | 2 | 3 | 1 | 0 |
| 207 | Noctuidae | *Resapamea vulpecula* | 0 | 7 | 0 | 0 | 0 | 0 | 3 | 1 | 0 | 0 |
| 208 | Noctuidae | *Rhyacia ledereri* | 0 | 0 | 0 | 0 | 0 | 2 | 7 | 0 | 0 | 0 |
| 209 | Noctuidae | *Rhyacia lucifera* | 1 | 0 | 0 | 0 | 0 | 0 | 0 | 0 | 0 | 0 |
| 210 | Noctuidae | *Rhyacia simulans* | 3 | 0 | 0 | 0 | 2 | 54 | 16 | 6 | 1 | 0 |
| 211 | Noctuidae | *Sideridis egena* | 1 | 0 | 0 | 5 | 13 | 13 | 27 | 7 | 6 | 1 |
| 212 | Noctuidae | *Sideridis kitti* | 0 | 0 | 0 | 0 | 0 | 0 | 6 | 11 | 0 | 3 |
| 213 | Noctuidae | *Sideridis turbida* | 0 | 0 | 0 | 0 | 0 | 1 | 2 | 0 | 0 | 0 |
| 214 | Noctuidae | *Simyra nervosa* | 0 | 1 | 0 | 0 | 0 | 0 | 1 | 19 | 0 | 0 |
| 215 | Noctuidae | *Syngrapha ain* | 0 | 0 | 0 | 0 | 0 | 0 | 1 | 0 | 0 | 6 |
| 216 | Noctuidae | *Xestia kollari* | 0 | 0 | 0 | 0 | 0 | 1 | 0 | 0 | 1 | 1 |
| 217 | Noctuidae | *Xestia versuta* | 0 | 0 | 0 | 0 | 1 | 0 | 0 | 1 | 0 | 0 |
| 218 | Notodontidae | *Clostera albosigma* | 0 | 0 | 0 | 0 | 0 | 0 | 0 | 0 | 2 | 9 |
| 219 | Notodontidae | *Clostera anachoreta* | 0 | 0 | 0 | 0 | 0 | 0 | 0 | 0 | 0 | 1 |
| 220 | Notodontidae | *Furcula furcula* | 0 | 0 | 0 | 0 | 0 | 0 | 0 | 0 | 0 | 1 |
| 221 | Notodontidae | *Nerice davidi* | 0 | 0 | 0 | 0 | 0 | 0 | 0 | 1 | 0 | 1 |
| 222 | Notodontidae | *Notodontidae* sp1 | 0 | 0 | 0 | 1 | 0 | 2 | 0 | 0 | 0 | 0 |
| 223 | Notodontidae | *Phalera bucephala* | 0 | 0 | 0 | 0 | 0 | 0 | 0 | 0 | 1 | 0 |
| 224 | Notodontidae | *Ptilodon kuwayamae* | 0 | 0 | 0 | 0 | 0 | 0 | 0 | 1 | 0 | 0 |
| 225 | Sessidae | *Sessidae* sp*1* | 0 | 0 | 0 | 0 | 0 | 0 | 2 | 0 | 0 | 0 |
| 226 | Sphingidae | *Callambulyx tatarinovii eversmanni* | 0 | 0 | 0 | 0 | 0 | 0 | 0 | 2 | 0 | 5 |
| 227 | Sphingidae | *Deiliphila* sp | 0 | 0 | 0 | 0 | 0 | 0 | 1 | 1 | 0 | 0 |
| 228 | Sphingidae | *Hyles chuvilini* | 5 | 14 | 0 | 3 | 0 | 0 | 1 | 1 | 0 | 0 |
| 229 | Sphingidae | *Hyles gallii* | 0 | 0 | 0 | 0 | 0 | 2 | 41 | 18 | 17 | 22 |
| 230 | Sphingidae | *Hyles hippophaes* | 0 | 1 | 0 | 0 | 0 | 0 | 0 | 0 | 0 | 0 |
| 231 | Sphingidae | *Laothoe amurensis* | 0 | 0 | 0 | 0 | 0 | 0 | 5 | 11 | 17 | 8 |
| 232 | Sphingidae | *Marumba gaschkewitschii* | 0 | 0 | 0 | 0 | 0 | 0 | 0 | 0 | 0 | 2 |
| 233 | Sphingidae | *Smerinthus caecus* | 0 | 0 | 0 | 0 | 0 | 0 | 0 | 1 | 1 | 8 |
| 234 | Sphingidae | *Sphinx ligustri* | 0 | 0 | 0 | 0 | 0 | 0 | 0 | 13 | 1 | 0 |
| 235 | Sphingidae | *Sphinx morio* | 0 | 0 | 0 | 1 | 0 | 0 | 2 | 1 | 0 | 0 |
| 236 | Zygaenidae | *Jordanita* sp1*.* | 0 | 0 | 0 | 0 | 0 | 0 | 0 | 0 | 2 | 0 |

Table S3 Species diversity (Hill numbers) and abundance along the transect from south (#1) to north (#10). The high individual numbers of sites 3, 4 and 5 are due to the high abundance of *Agrotis ripae.*

| **Site** | **Species richness** | **Shannon diversity** | **Simpson diversity** | **Abundance** |
| --- | --- | --- | --- | --- |
| 1 | 38 | 7.54 | 4. | 866 |
| 2 | 35 | 16.28 | 278.30 | 157 |
| 3 | 12 | 1.31 | 1.10 | 1516 |
| 4 | 27 | 1.71 | 1.25 | 1925 |
| 5 | 32 | 1.89 | 1.30 | 2752 |
| 6 | 35 | 14.12 | 9.63 | 328 |
| 7 | 87 | 31.90 | 16.58 | 646 |
| 8 | 118 | 45.50 | 21.00 | 866 |
| 9 | 96 | 28.94 | 14.73 | 937 |
| 10 | 100 | 37.76 | 22.14 | 1105 |

Table S4 The results of GLMs comparing the species richness of four main families or family groups (Noctuidae, Geometridae, Erebidae and Others) among ten study sites along the latitudinal gradients.

| **Family** | **Site** | **Estimate** | **Standard error** | ***z*- value** | ***p* value** | ***p* adjusted** |
| --- | --- | --- | --- | --- | --- | --- |
| Noctuidae | (Intercept) | 1.85629799 | 0.176776682 | 10.50080795 | 8.56E-26 | 8.56E-25 |
| Noctuidae | Site2 | 0.916290732 | 0.228217722 | 4.014985002 | 5.94E-05 | **0.000297248** |
| Noctuidae | Site3 | -0.121696935 | 0.300122516 | -0.405490852 | 0.685116741 | 0.685116741 |
| Noctuidae | Site4 | 0.377294231 | 0.258774576 | 1.458003476 | 0.144839585 | 0.28967917 |
| Noctuidae | Site5 | 0.682675881 | 0.239928708 | 2.845328042 | 0.004436571 | **0.013309712** |
| Noctuidae | Site6 | 0.806289837 | 0.233464793 | 3.453582135 | 0.000553194 | **0.002212775** |
| Noctuidae | Site7 | 1.544899391 | 0.205818139 | 7.506138165 | 6.09E-14 | **4.87E-13** |
| Noctuidae | Site8 | 1.679818709 | 0.202382641 | 8.300211437 | 1.04E-16 | **9.35E-16** |
| Noctuidae | Site9 | 1.349154814 | 0.211573885 | 6.376754922 | 1.81E-10 | **1.09E-09** |
| Noctuidae | Site10 | 1.362577835 | 0.211147647 | 6.453199244 | 1.10E-10 | **7.67E-10** |
| Erebidae | (Intercept) | 1.16315081 | 0.249999776 | 4.652607403 | 3.28E-06 | **2.95E-05** |
| Erebidae | Site2 | -0.064538521 | 0.478713265 | -0.134816655 | 0.892756837 | 1 |
| Erebidae | Site3 | -0.470003629 | 0.749999925 | -0.626671568 | 0.530874545 | 1 |
| Erebidae | Site4 | -0.470003629 | 0.749999925 | -0.626671568 | 0.530874545 | 1 |
| Erebidae | Site5 | 0.223143551 | 0.433012573 | 0.515328111 | 0.606323758 | 1 |
| Erebidae | Site6 | -0.315852949 | 0.453163358 | -0.696995783 | 0.485805433 | 1 |
| Erebidae | Site7 | 0.682675881 | 0.339310263 | 2.01195176 | 0.044225022 | 0.265350134 |
| Erebidae | Site8 | 0.916290732 | 0.322748422 | 2.839024669 | 0.004525166 | **0.036201327** |
| Erebidae | Site9 | 0.733969175 | 0.33540985 | 2.188275553 | 0.028649537 | 0.200546756 |
| Erebidae | Site10 | 1.499437017 | 0.292840745 | 5.120315535 | 3.05E-07 | **3.05E-06** |
| Geometridae | (Intercept) | 1.098612289 | 0.3333333 | 3.295837198 | 0.000981289 | **0.005887732** |
| Geometridae | Site2 | -1.86E-14 | 0.66666665 | -2.80E-14 | 1 | 1 |
| Geometridae | Site3 | -0.405465108 | 0.600925194 | -0.674734746 | 0.499844305 | 1 |
| Geometridae | Site4 | -0.405465108 | 0.600925194 | -0.674734746 | 0.499844305 | 1 |
| Geometridae | Site5 | -0.405465108 | 0.781735946 | -0.518672719 | 0.603988989 | 1 |
| Geometridae | Site6 | 0.287682072 | 0.440957994 | 0.652402443 | 0.514141589 | 1 |
| Geometridae | Site7 | 1.299282984 | 0.37605057 | 3.455075159 | 0.000550139 | **0.003850976** |
| Geometridae | Site8 | 1.863218433 | 0.358263126 | 5.200698308 | 1.99E-07 | **1.99E-06** |
| Geometridae | Site9 | 1.828127113 | 0.359121472 | 5.090553634 | 3.57E-07 | **3.21E-06** |
| Geometridae | Site10 | 1.714798428 | 0.362092652 | 4.735800127 | 2.18E-06 | **1.75E-05** |
| Other | (Intercept) | 1.029619417 | 0.267261221 | 3.852483396 | 0.000116926 | **0.001052333** |
| Other | Site2 | 0.174353387 | 0.414039322 | 0.42110345 | 0.673679542 | 1 |
| Other | Site4 | 0.356674944 | 0.39339788 | 0.906651922 | 0.364590895 | 1 |
| Other | Site5 | 0.223143551 | 0.462908852 | 0.482046412 | 0.629772976 | 1 |
| Other | Site6 | 0.47445798 | 0.427246617 | 1.110501432 | 0.266783011 | 1 |
| Other | Site7 | 0.762140052 | 0.356348307 | 2.13875031 | 0.032455896 | 0.162279479 |
| Other | Site8 | 1.203972804 | 0.327326818 | 3.678197864 | 0.000234888 | **0.001879102** |
| Other | Site9 | 0.816207273 | 0.352221719 | 2.317311027 | 0.020486795 | 0.12292077 |
| Other | Site10 | 1.049822125 | 0.336296211 | 3.121718561 | 0.001797987 | **0.012585911** |

Table S5 The results of GLMs comparing the Shannon diversity of four main families or family groups (Noctuidae, Geometridae, Erebidae and Other) among ten study sites along the latitudinal gradients.

| **Family** | **Site** | **Estimate** | **Standard error** | ***t*-value** | ***p* value** | ***p* adjusted** |
| --- | --- | --- | --- | --- | --- | --- |
| Noctuidae | (Intercept) | 4.018823734 | 1.071812393 | 3.749558932 | 0.001108734 | **0.007761139** |
| Noctuidae | Site2 | 2.684777491 | 1.750262309 | 1.533928644 | 0.139303614 | 0.557214456 |
| Noctuidae | Site3 | -2.205656746 | 1.750262309 | -1.260186393 | 0.220805727 | 0.565562197 |
| Noctuidae | Site4 | -2.375224938 | 1.750262309 | -1.357067981 | 0.188520732 | 0.565562197 |
| Noctuidae | Site5 | -1.809126987 | 1.750262309 | -1.033631917 | 0.312537656 | 0.565562197 |
| Noctuidae | Site6 | 4.702787492 | 1.750262309 | 2.686904396 | 0.013468327 | 0.067341636 |
| Noctuidae | Site7 | 10.73924122 | 1.750262309 | 6.135789571 | 3.55E-06 | **3.20E-05** |
| Noctuidae | Site8 | 15.50300473 | 1.750262309 | 8.857532183 | 1.05E-08 | **1.05E-07** |
| Noctuidae | Site9 | 6.388437072 | 1.750262309 | 3.649988369 | 0.001410688 | **0.008464125** |
| Noctuidae | Site10 | 8.243544979 | 1.750262309 | 4.709891161 | 0.000106597 | **0.00085278** |
| Erebidae | (Intercept) | 2.68730168 | 0.466558516 | 5.759838452 | 2.93E-05 | **0.000263478** |
| Erebidae | Site2 | -0.93349506 | 0.872851059 | -1.069478063 | 0.300724039 | 1 |
| Erebidae | Site3 | -1.151084494 | 1.142830299 | -1.00722259 | 0.328818344 | 1 |
| Erebidae | Site4 | -1.180308614 | 1.142830299 | -1.032794296 | 0.317059676 | 1 |
| Erebidae | Site5 | -0.40381132 | 0.872851059 | -0.462634852 | 0.649855592 | 1 |
| Erebidae | Site6 | -1.347267728 | 0.761886866 | -1.768330429 | 0.096064532 | 0.768516259 |
| Erebidae | Site7 | 0.0127062 | 0.761886866 | 0.01667728 | 0.986900267 | 1 |
| Erebidae | Site8 | 1.168565858 | 0.761886866 | 1.533778715 | 0.144618856 | 1 |
| Erebidae | Site9 | -0.14901377 | 0.761886866 | -0.195585167 | 0.847397153 | 1 |
| Erebidae | Site10 | 5.528386192 | 0.761886866 | 7.256177313 | 1.92E-06 | **1.92E-05** |
| Geometridae | (Intercept) | 2.378634425 | 1.150874497 | 2.066806095 | 0.057766063 | 0.346596381 |
| Geometridae | Site2 | -0.397362736 | 2.301748994 | -0.17263513 | 0.865409123 | 1 |
| Geometridae | Site3 | -0.741620894 | 1.819692356 | -0.407552898 | 0.689765612 | 1 |
| Geometridae | Site4 | -0.753377596 | 1.819692356 | -0.414013717 | 0.68513697 | 1 |
| Geometridae | Site5 | -1.067111011 | 2.301748994 | -0.463608766 | 0.650053762 | 1 |
| Geometridae | Site6 | -0.00673734 | 1.627582322 | -0.004139477 | 0.996755592 | 1 |
| Geometridae | Site7 | 3.839320868 | 1.627582322 | 2.358910401 | 0.03339124 | 0.233738677 |
| Geometridae | Site8 | 6.218089414 | 1.627582322 | 3.820445411 | 0.001873646 | **0.016862815** |
| Geometridae | Site9 | 6.925544723 | 1.627582322 | 4.255111787 | 0.000800173 | **0.00800173** |
| Geometridae | Site10 | 6.042500744 | 1.627582322 | 3.712562284 | 0.00231893 | **0.018551441** |
| Other | (Intercept) | 2.264481336 | 0.371521279 | 6.095159192 | 9.28E-06 | **7.80E-05** |
| Other | Site2 | 0.367824552 | 0.606691707 | 0.606279181 | 0.551899495 | 1 |
| Other | Site4 | 0.018069089 | 0.606691707 | 0.029782984 | 0.976567873 | 1 |
| Other | Site5 | -0.372104299 | 0.695052668 | -0.535361298 | 0.598952864 | 1 |
| Other | Site6 | -0.237192791 | 0.695052668 | -0.34125873 | 0.736860088 | 1 |
| Other | Site7 | 0.940955031 | 0.606691707 | 1.55096076 | 0.138315225 | 0.691576123 |
| Other | Site8 | 3.059250909 | 0.606691707 | 5.042513143 | 8.47E-05 | **0.000592815** |
| Other | Site9 | 1.252460497 | 0.606691707 | 2.064410115 | 0.053695472 | 0.322172835 |
| Other | Site10 | 3.718189628 | 0.606691707 | 6.128631036 | 8.67E-06 | **7.80E-05** |

Table S6 The results of GLMs comparing the abundance of four main families or family groups (Noctuidae, Geometridae, Erebidae and Other) among ten study sites along the latitudinal gradients.

| **Family** | **Site** | **Estimate** | **Standard error** | ***z*-value** | ***p* value** | ***p* adjusted** |
| --- | --- | --- | --- | --- | --- | --- |
| Noctuidae | (Intercept) | 1.946824341 | 0.6075589 | 3.204338443 | 0.004090053 | **0.032336915** |
| Noctuidae | Site2 | 2.693952828 | 0.99213953 | 2.715296333 | 0.012636877 | **0.043430291** |
| Noctuidae | Site3 | 3.184113536 | 0.99213953 | 3.209340461 | 0.004042114 | **0.032336915** |
| Noctuidae | Site4 | 4.472266794 | 0.99213953 | 4.50769943 | 0.000174559 | **0.001745585** |
| Noctuidae | Site5 | 4.204149544 | 0.99213953 | 4.237457955 | 0.000337826 | **0.00304043** |
| Noctuidae | Site6 | 2.399421745 | 0.99213953 | 2.418431756 | 0.024311682 | **0.043430291** |
| Noctuidae | Site7 | 3.037141112 | 0.99213953 | 3.061203611 | 0.005720323 | **0.034321937** |
| Noctuidae | Site8 | 2.760652456 | 0.99213953 | 2.782524406 | 0.010857573 | **0.043430291** |
| Noctuidae | Site9 | 2.551077272 | 0.99213953 | 2.571288811 | 0.017416684 | **0.043430291** |
| Noctuidae | Site10 | 2.991055428 | 0.99213953 | 3.014752802 | 0.006372817 | **0.034321937** |
| Erebidae | (Intercept) | 0.895467363 | 0.460422151 | 1.944883322 | 0.069581159 | 0.487068112 |
| Erebidae | Site2 | 1.029606438 | 0.861370971 | 1.195311279 | 0.249382115 | 1 |
| Erebidae | Site3 | -0.202320182 | 1.127799337 | -0.179393777 | 0.859880658 | 1 |
| Erebidae | Site4 | -0.202320182 | 1.127799337 | -0.179393777 | 0.859880658 | 1 |
| Erebidae | Site5 | 1.592899508 | 0.861370971 | 1.849260726 | 0.082981934 | 0.497891601 |
| Erebidae | Site6 | -0.664418303 | 0.751866224 | -0.88369218 | 0.389947955 | 1 |
| Erebidae | Site7 | 0.952925119 | 0.751866224 | 1.267413122 | 0.223143954 | 1 |
| Erebidae | Site8 | 2.117479322 | 0.751866224 | 2.81629797 | 0.012416593 | 0.099332747 |
| Erebidae | Site9 | 2.95526726 | 0.751866224 | 3.930575898 | 0.001194334 | **0.010749002** |
| Erebidae | Site10 | 3.420869134 | 0.751866224 | 4.549837488 | 0.000327978 | **0.003279781** |
| Geometridae | (Intercept) | 1.059351277 | 0.435129871 | 2.43456344 | 0.028888597 | 0.144442987 |
| Geometridae | Site2 | -0.366204096 | 0.870259743 | -0.420798617 | 0.68029024 | 1 |
| Geometridae | Site3 | -0.366204096 | 0.688000736 | -0.532272826 | 0.602882648 | 1 |
| Geometridae | Site4 | -0.163471542 | 0.688000736 | -0.237603732 | 0.815630453 | 1 |
| Geometridae | Site5 | -1.059351277 | 0.870259743 | -1.21728172 | 0.243618489 | 0.974473958 |
| Geometridae | Site6 | 1.844463163 | 0.615366566 | 2.997340555 | 0.009601992 | 0.057611953 |
| Geometridae | Site7 | 2.305351361 | 0.615366566 | 3.746305844 | 0.002169175 | **0.015184228** |
| Geometridae | Site8 | 3.620061931 | 0.615366566 | 5.882773186 | 3.99E-05 | **0.000338417** |
| Geometridae | Site9 | 3.640710093 | 0.615366566 | 5.916327433 | 3.76E-05 | **0.000338417** |
| Geometridae | Site10 | 3.735866589 | 0.615366566 | 6.070961274 | 2.88E-05 | **0.000288195** |
| Other | (Intercept) | 1.105885818 | 0.301859394 | 3.663579264 | 0.001777093 | **0.009798584** |
| Other | Site2 | 0.85614886 | 0.492934327 | 1.73684163 | 0.099497268 | 0.397989074 |
| Other | Site4 | 0.258895703 | 0.492934327 | 0.525213379 | 0.605844623 | 1 |
| Other | Site5 | -0.210006083 | 0.564727216 | -0.371871723 | 0.714330007 | 1 |
| Other | Site6 | 0.248139283 | 0.564727216 | 0.439396714 | 0.665603745 | 1 |
| Other | Site7 | 1.824609525 | 0.492934327 | 3.701526605 | 0.001633097 | **0.009798584** |
| Other | Site8 | 2.320965245 | 0.492934327 | 4.708467475 | 0.000175171 | **0.001576537** |
| Other | Site9 | 1.878315974 | 0.492934327 | 3.810479151 | 0.001281121 | **0.008967846** |
| Other | Site10 | 1.915500136 | 0.492934327 | 3.885913463 | 0.001082882 | **0.008663054** |

Table S7 Livestock numbers of each site. Data are obtained for each site from the National Statistical Office of Mongolia ^26^

| Site | Year | Camel | Cow | Goat | Horse | Sheep |
| --- | --- | --- | --- | --- | --- | --- |
| 1 | 2018 | 470 | 980 | 44280 | 2390 | 10370 |
| 1 | 2019 | 450 | 1080 | 49590 | 2680 | 11400 |
| 2 | 2018 | 380 | 250 | 7230 | 330 | 2760 |
| 2 | 2019 | 470 | 270 | 9740 | 400 | 3480 |
| 3 | 2018 | 4100 | 580 | 37300 | 1670 | 20360 |
| 3 | 2019 | 4280 | 630 | 39750 | 1880 | 21760 |
| 4 | 2018 | 5340 | 360 | 36740 | 3890 | 40860 |
| 4 | 2019 | 5540 | 370 | 37620 | 3950 | 39890 |
| 5 | 2018 | 80 | 1610 | 37230 | 6220 | 45050 |
| 5 | 2019 | 70 | 1550 | 38860 | 6450 | 47320 |
| 6 | 2018 | 320 | 3860 | 48170 | 7720 | 63180 |
| 6 | 2019 | 350 | 3610 | 50350 | 8120 | 64670 |
| 7 | 2018 | 0 | 19580 | 13310 | 5680 | 16800 |
| 7 | 2019 | 0 | 21200 | 15320 | 5970 | 16710 |
| 8 | 2018 | 0 | 4730 | 3830 | 2570 | 3230 |
| 8 | 2019 | 0 | 5500 | 5190 | 2580 | 3960 |
| 9 | 2018 | 0 | 4280 | 16210 | 3320 | 21000 |
| 9 | 2019 | 0 | 5390 | 20730 | 3870 | 25290 |
| 10 | 2018 | 0 | 6040 | 9700 | 2400 | 11310 |
| 10 | 2019 | 0 | 6200 | 11410 | 2610 | 12030 |

Table S8 Model parameters for all fitted models of relevant environmental variables for species richness, Shannon diversity, and Simpson diversity. For each environmental variables three types of model are fitted: environmental variable as a single factor, additive: environmental variable+biome (B), interaction effect: environmental variable x biome (B). For each model, model estimate- *β,* standard error-St.err., *p* value, and AIC are given in the table. The best model with the lowest AIC is marked in bold. Abbreviations of the environmental variables are as follows: P – precipitation, B- biome, Veg cov – vegetation cover, L - livestock, W – wind, A- altitude, Veg rich- vegetation richness.

|  | **Model for richness** | | | | **Model for Shannon diversity** | | | | **Model for Simpson diversity** | | | |
| --- | --- | --- | --- | --- | --- | --- | --- | --- | --- | --- | --- | --- |
| Predictor | *β* | St.err | *p*-value | AIC | *β* | St.err | *p*-value | AIC | *β* | St.err | *p*-value | AIC |
| Biome |  |  |  | 306.940 | 1.639 | 0.21 | *** | 84.732 | 1.400 | 0.192 | *** | 77.364 |
| P | 0.010 | 0.001 | *** | **294.470** | 0.012 | 0.001 | *** | **66.805** | 0.010 | 0.001 | *** | **62.341** |
| P+B |  |  |  | 296.370 |  |  |  | 68.805 |  |  |  | 64.341 |
| *Precipitation* | 0.010 | 0.002 | *** |  | 0.012 | 0.003 | *** |  | 0.010 | 0.003 | *** |  |
| *Biome* | -0.121 | 0.369 | ns |  | -0.003 | 0.397 | ns |  | 0.007 | 0.376 | ns |  |
| P x B | 0.002 | 0.005 | ns | 298.260 | -0.004 | 0.005 | ns | 70.261 | -0.002 | 0.005 | ns | 66.198 |
| Veg cov | 0.020 | 0.005 | *** | 324.720 | 0.029 | 0.005 | *** | 100.767 | 0.026 | 0.005 | *** | 88.388 |
| Veg_cov+B |  |  |  | **305.670** |  |  |  | **77.385** |  |  |  | **68.146** |
| *Vegetation cover* | 0.008 | 0.004 | ns |  | 0.015 | 0.005 | ** |  | 0.014 | 0.004 | ** |  |
| *Biome* | 1.098 | 0.209 | *** |  | 1.274 | 0.223 | *** |  | 1.040 | 0.198 | *** |  |
| Veg_cov x B | -0.006 | 0.009 | ns | 307.240 | -0.011 | 0.010 | ns | 77.950 | -0.010 | 0.009 | ns | 68.824 |
| L | 0.000 | 0.000 | ** | 328.890 | 0.000 | 0.000 | *** | 109.965 | 0.000 | 0.000 | *** | 97.140 |
| L + B |  |  |  | 305.670 |  |  |  | **59.707** |  |  |  | 48.121 |
| *Livestock* | -8E-06 | 2E-06 | ** |  | -1E-05 | 2E-06 | *** |  | -1E-05 | 2E-06 | *** |  |
| *Biome* | 1E+00 | 2E-01 | *** |  | 2E+00 | 2E-01 | *** |  | 1E+00 | 1E-01 | *** |  |
| L x B | -1E-05 | 5E-06 | * | **299.670** | 4E-06 | 5E-06 | ns | 60.776 | 6E-06 | 4E-06 | ns | **47.867** |
| W | -9E-01 | 1E-01 | *** | **299.040** | -1E+00 | 0.134 | *** | 84.733 | -9E-01 | 1E-01 | *** | 78.954 |
| W + B |  |  |  | 300.780 |  |  |  | 84.698 |  |  |  | 78.351 |
| *Wind* | -1.0E+00 | 3.0E-01 | *** |  | -0.539 | 0.388 | ns |  | -0.349 | 0.358 | ns |  |
| *Biome* | -2.6E-01 | 4.8E-01 | ns |  | 0.845 | 0.608 | ns |  | 0.886 | 0.562 | ns |  |
| W x B | -1.2E+00 | 7.0E-01 | ns | 299.750 | -2.800 | 0.759 | *** | **73.862** | -2.790 | 0.679 | *** | **64.968** |
| A | -1.1E-03 | 2.5E-04 | *** | 320.410 | -0.001 | 0.000 | *** | 110.627 | -0.001 | 0.0003 | ** | 101.310 |
| A + B |  |  |  | 308.500 |  |  |  | 86.575 |  |  |  | 79.127 |
| *Altitude* | -0.0002 | 0.0003 |  |  | 0.0001 | 0.0003 | ns |  | 0.000 | 0.0003 | ns |  |
| *Biome* | 1.152 | 0.256 | *** |  | 1.717 | 0.295 | *** |  | 1.487 | 0.268 | *** |  |
| A x B | -0.001 | 0.001 | * | **304.940** | -0.002 | 0.001 | *** | **76.427** | -0.002 | 0.0005 | *** | **68.857** |
| Veg rich | 0.065 | 0.009 | *** | 306.590 | 0.072 | 0.011 | *** | 94.650 | 0.063 | 0.010 | *** | 83.678 |
| Veg rich+B |  |  |  | **296.890** |  |  |  | **77.600** |  |  |  | **69.472** |
| *Vegitation richness* | 0.039 | 0.010 | *** |  | 0.036 | 0.012 | ** |  | 0.034 | 0.011 | ** |  |
| *Biome* | 0.773 | 0.204 | *** |  | 1.163 | 0.245 | *** |  | 0.221 | 0.221 | *** |  |
| Veg rich x B | -0.024 | 0.025 | ns | 298.010 | 0.019 | 0.028 | ns | 79.070 | 0.028 | 0.025 | ns | 70.073 |


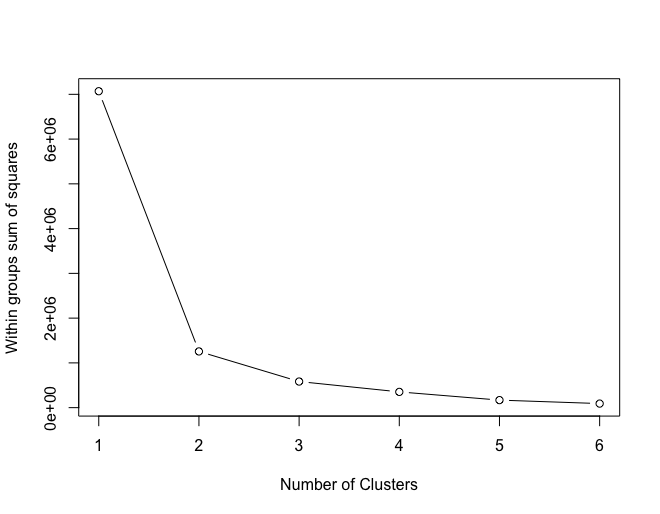
 Figure S1 Scree plot shows the suitable number of clusters for ten sites. Optimal number of clusters corresponds to the elbow location (2), thus indicating two groups, southern (desert) and northern sites (grassland).


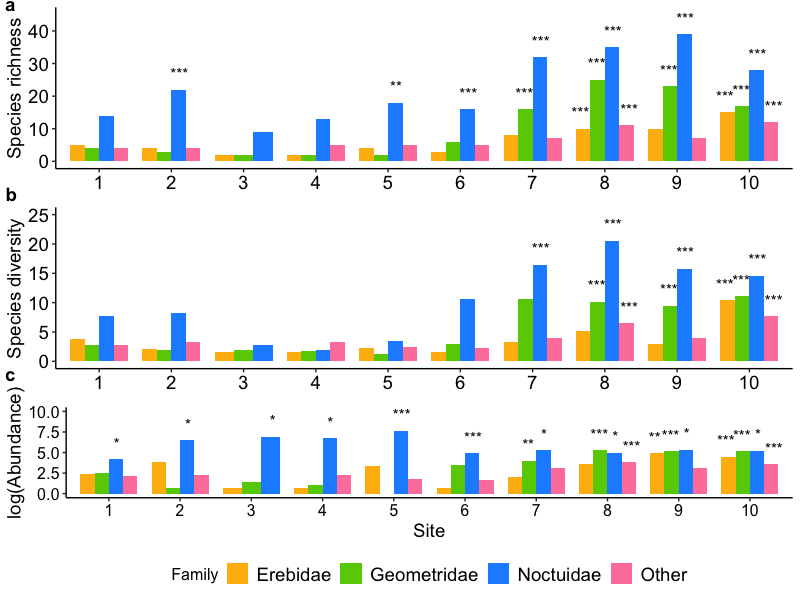


Figure S2 Species richness, species diversity and abundance (Hill numbers) of Noctuidae, Geometridae, Erebidae and other families of each site. (Compared with GLM).


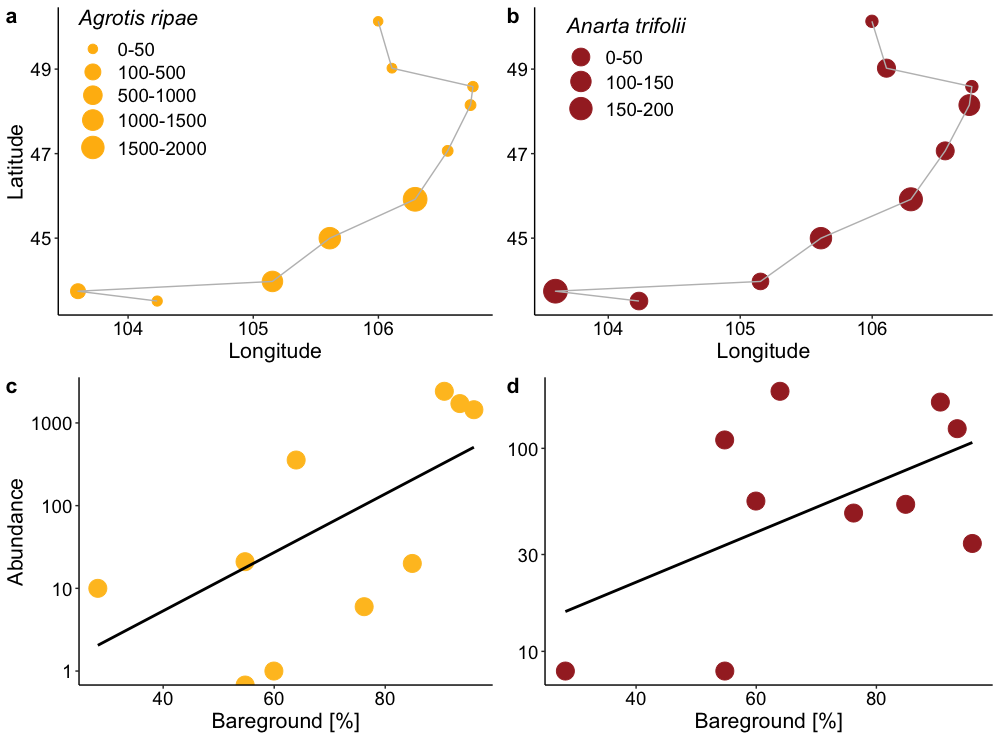


Figure S3 Abundance of two most dominant species *Agrotis ripae* (left) and *Anarta trifolii* (right) along the latitudinal gradient. a) Abundance of *Agrotis ripae*, b) Abundance of *Anarta trifolii*. Correlation of the percentage of Bareground with Abundance of c) *Agrotis ripae* and d) *Anarta trifolii* after log10 transformation. GLM line was fitted to each correlation graph to show the response of Abundance to Bareground.


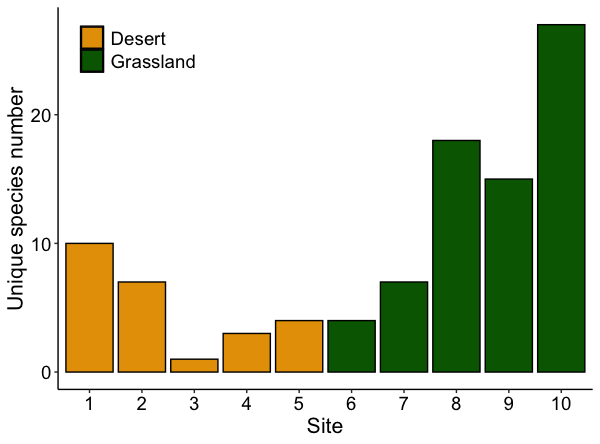


Figure S4 Number of unique species per site. These species occurred only in one site. Site three had only one unique species, whereas site 10 had 27 unique species.


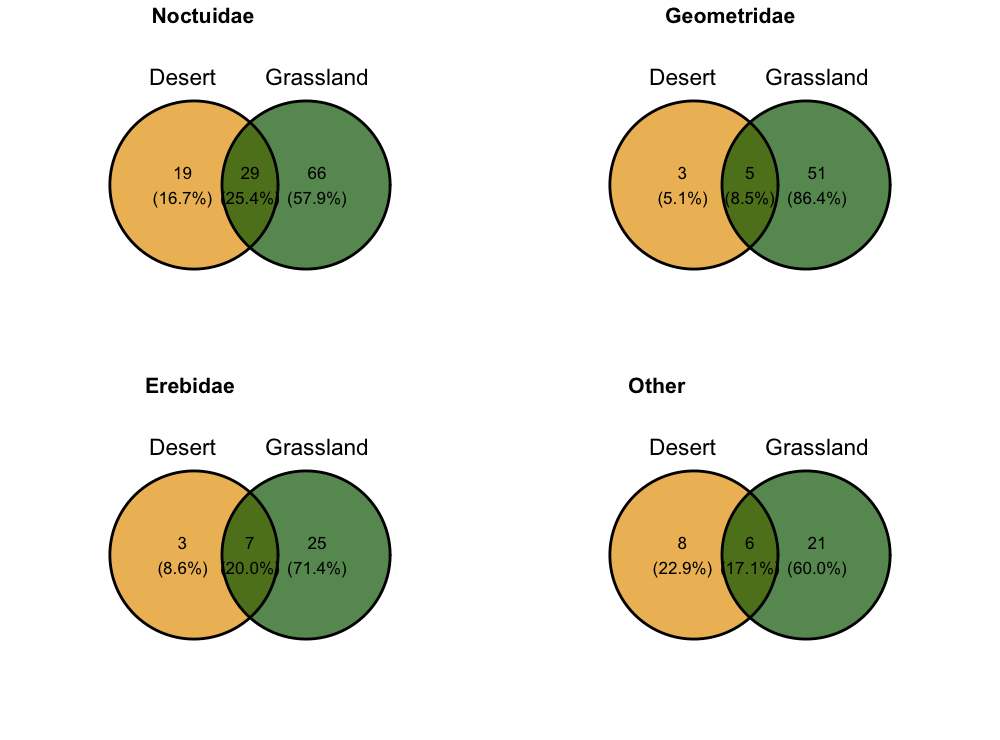
 Figure S5 Venn diagrams showing the species overlaps of assemblages by main groups. a) Noctuidae, b) Geometridae, c) Erebidae, and d) Other


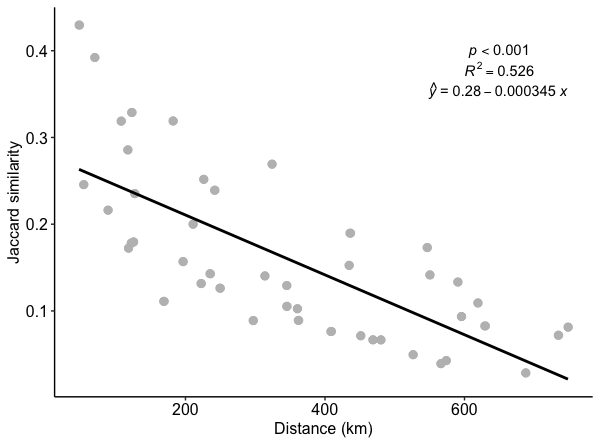


Figure S6 Distance-decay relationship for Mongolian moth assemblages. Shown is pairwise Jaccard similarity of all sites plotted against the pairwise distance in km.


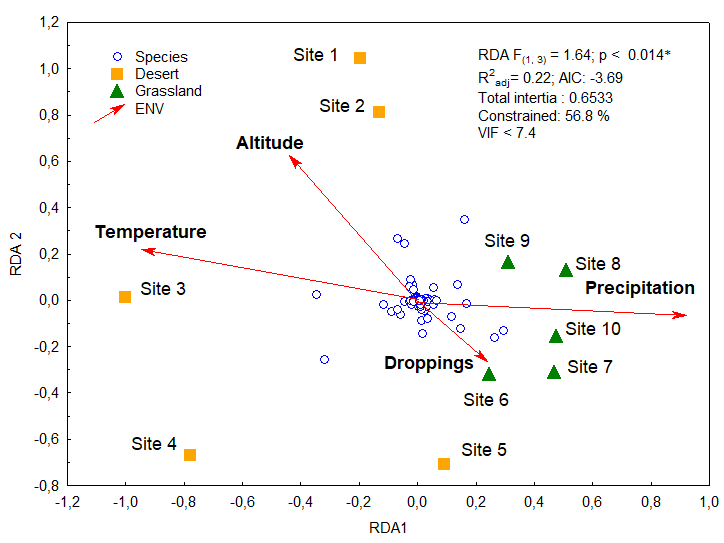


Figure S7 Parsimonious RDA of the plant communities. Four parameters explained 22% of the variation. Z-standardized environmental parameters had variance inflation factors (VIF) < 7.4. Species data were Hellinger-transformed prior to analysis. While site 5 is intermediate in plant species composition, the plots of plant communities in desert and grassland are clearly separated and differ significantly in RDA1 (U-test, U = -2.61, p < 0.009).


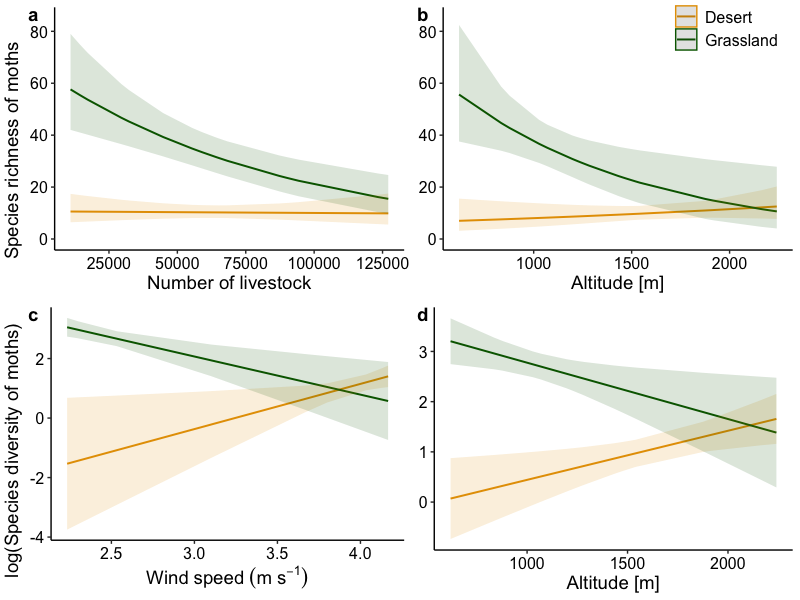


Figure S8 Interaction effects of environmental variables and biome type on the species richness and the diversity of macro-moths. a) Number of livestock x Biome, b) Altitude, c) Wind speed, d) Altitude.
